# Interactions of the α3β2 Nicotinic Acetylcholine Receptor Interfaces with α-Conotoxin LsIA and its Carboxylated C-terminus Analogue: Molecular Dynamics Simulations

**DOI:** 10.3390/md18070349

**Published:** 2020-07-03

**Authors:** Jierong Wen, David J. Adams, Andrew Hung

**Affiliations:** 1 School of Science, RMIT University, Melbourne, VIC 3000, Australia; 2 Illawarra Health and Medical Research Institute (IHMRI), University of Wollongong, Wollongong, NSW 2522, Australia; djadams@uow.edu.au

**Keywords:** MD simulation, homology modelling, α-conotoxin, nicotinic acetylcholine receptor interface, C-terminal amidation/carboxylation

## Abstract

Notably, α-conotoxins with carboxy-terminal (C-terminal) amidation are inhibitors of the pentameric nicotinic acetylcholine receptors (nAChRs), which are therapeutic targets for neurological diseases and disorders. The (α3)_2_(β2)_3_ nAChR subunit arrangement comprises a pair of α3(+)β2(−) and β2(+)α3(−) interfaces, and a β2(+)β2(−) interface. The β2(+)β2(−) interface has been suggested to have higher agonist affinity relative to the α3(+)β2(−) and β2(+)α3(−) interfaces. Nevertheless, the interactions formed by these subunit interfaces with α-conotoxins are not well understood. Therefore, in order to address this, we modelled the interactions between α-conotoxin LsIA and the α3β2 subtype. The results suggest that the C-terminal carboxylation of LsIA predominantly influenced the enhanced contacts of the conotoxin via residues P7, P14 and C17 on LsIA at the α3(+)β2(−) and β2(+)α3(−) interfaces. However, this enhancement is subtle at the β2(+)β2(−) site, which can compensate the augmented interactions by LsIA at α3(+)β2(−) and β2(+)α3(−) binding sites. Therefore, the divergent interactions at the individual binding interface may account for the minor changes in binding affinity to α3β2 subtype by C-terminal carboxylation of LsIA versus its wild type, as shown in previous experimental results. Overall, these findings may facilitate the development of new drug leads or subtype-selective probes.

## 1. Introduction

Neuronal nicotinic acetylcholine receptors (nAChRs) are ligand-gated ion channels [1] that play important roles in the mediating signalling transmission between neurons. Therefore, they are related to numerous neurological disorders and diseases, such as Parkinson’s disease, Alzheimer’s disease, dementia, schizophrenia and addiction [2,3,4]. These receptors exist as five subunit combinations of α subunit (α2-10) only (homomeric α7 and α9 subtypes), or heteromeric combinations of α and β (β2-4) subunits [5]. Each subunit comprises an N-terminal extracellular domain (ECD), transmembrane domain (TMD) and an intracellular domain (ICD). The ECD is of particular importance due to the presence of the canonical ligand-binding domain (LBD) at the interface of two adjacent subunits [6,7]. It has been demonstrated that the α subunit ECD can facilitate the binding of agonists [8], such as acetylcholine (ACh), at the LBD [1], resulting in the opening of the channel in the TMD. Moreover, neuronal nAChRs are widely recognised as a target of α-conotoxins, such as LsIA, showing high potency at α3β2 (IC_50_ 10.3 nM) and α7 (IC_50_ 10 nM) nAChR subtypes [9]. 

The α3β2 subtype is expressed in the central and peripheral nervous systems, including the cerebellum, spinal cord, and autonomic ganglion neurons [10,11]. Various stoichiometries of the α3 and β2 subunits are known to exist, with the most common consisting of two α3 and three β2 subunits, ((α3)_2_(β2)_3_) [12]. A recent study has suggested that the (α3)_3_(β2)_2_ stoichiometry may also exist at the motoneuron-Renshaw cell synapse in the spinal cord [13]. In this stoichiometry, a pair of β2(+)α3(−) and α3(+)β2(−) interfaces are commensurate with those constituting the (α3)_2_(β2)_3_ isoform. However, an α3(+)α3(−) interface in the former, (α3)_3_(β2)_2_, and a β2(+)β2(−) interface in the latter, (α3)_2_(β2)_3_, cause the main structural differences between the α3β2 isoforms. Moreover, the physiological and pharmacological properties of various nAChR isoforms may be affected by the ratio of α to β subunits [14,15]. In addition, previous studies have also demonstrated the existence of alternative stoichiometries of nAChRs, such as α4β2 ((α4)_3_(β2)_2_ and (α4)_2_(β2)_3_), α3β4 ((α3)_3_(β4)_2_ and (α3)_2_(β4)_3_) and α9α10 nAChR ((α9)_3_(α10)_2_ and (α9)_2_(α10)_3_) [14,15,16,17,18,19].

More importantly, a previous study by Boffi et al. has demonstrated that the different combinations of subunit interfaces of α9α10 nAChR showed distinct contributions of α9 and α10, depending on which serves as the (−) face to the binding of ACh [18]. Furthermore, the presence of α-conotoxins targeting multiple interfaces of nAChRs has attracted attention for investigating the binding determinants of the successful binding of the ligand at the receptors [15,19]. However, the role of the different interfaces in the binding of LsIA to nAChRs remains unclear.

The combinations of an α subunit as the principal (+) face with one β subunit as complementary (−) face that forms interfaces which are commonly accepted as the canonical region binding agonists and antagonists. However, other different interfaces of α3β2 nAChR, namely β2(+)β2(−), β2(+)α3(−) and α3(+)α3(−), may also contribute to the binding of ligands, such as ACh and α-conotoxin MII [20]. Further, the residues on β subunits have been suggested to be involved in the nAChR subtype selectivity of α-conotoxins [21,22,23]. Other studies emphasized the important role of the β subunit as a structural component, which serves as an accessory binding face for α subunits [18,21,24,25]. Therefore, it is intriguing to explore the role of the β2 subunit in interacting with novel α-conotoxins at the different binding interfaces of the (α3)_2_(β2)_3_ nAChR isoform. 

LsIA, isolated from the venom of *Conus limpusi*, is known to inhibit rat α3β2 nAChR with nanomolar potency [9]. Similar to a range of α-conotoxins, native LsIA possesses an amidated C-terminus (C-T) and two disulfide bridges contributed by four conserved cysteine residues (Cys1-Cys3, Cys2-Cys4). These disulfide bridges also confer it with two structural loops consistent with the 4/7 subclass of α-conotoxins, characterized by four amino acid residues in the first loop (loop 1 in the present study) and seven residues in the second loop (loop 2) [21,26]. 

These structural characteristics may contribute to the high binding affinity of α-conotoxins to nAChR subtypes. For example, LsIA has been shown to selectively inhibit human α7 and rat α3β2 nAChRs expressed in *Xenopus laevis* oocytes [9,27], whereas the LsIA[R10F] mutant preferentially binds at human α3β4 nAChR [27]. Furthermore, the carboxy-terminal (C-terminal) amidation, together with the sulfated tyrosine, are crucial for the activity of AnIB on α7, rather than α3β2 [28]. Similar to native LsIA, most 4/7 α-conotoxins possess an amidated C-T in their natural form, namely α-conotoxins Vc1.1, MII, GIC, AnIB, and TxIA. However, an amidated C-T does not exist in certain identified α-conotoxins, such as GID and RgIA with a C-terminal carboxylate, that also bind at α3β2, α7 and α9α10 nAChRs with high potency [26,29]. Notably, previous experimental results have shown that the C-terminal carboxylation enhanced the binding affinity of LsIA at rat α3β2 nAChR three-fold, yet this modification (C-terminal carboxylation of LsIA) resulted in a three-fold reduced affinity at human α7 nAChR. In the present context, “C-terminal carboxylation” or “carboxylated C-terminus” refers to the presence of a single COO^-^ functional group that terminates the peptide backbone, compatible with the terminology adopted by Inserra et al. [9] Although these differences are marginal, the investigation of the effects of C-T of α-conotoxins on receptor interface interactions may nonetheless shed light on the possibilities for improvements and design of α-conotoxins with high selectivity to individual nAChR subtypes [30]. 

The determined crystal structure of the acetylcholine binding protein (AChBP) has facilitated the in silico studies of conotoxin-nAChR complexes. It has been shown that residues at the LBD of the ECD of α3β2 and α7 share high sequence similarity with those of AChBP [31], which can provide reliable homology models of nAChRs for molecular dynamics (MD) simulations and subsequent computational analyses. Previous studies have employed the molecular modelling of nAChRs based on the crystal structures of AChBP [31,32] and α1 nAChR subunit [33]. Moreover, numerous studies have produced comparative models of conotoxins binding at nAChRs complex, built using the co-crystal structures of *Aplysia californica* (Ac) AChBP with α-conotoxins TxIA[A10L], PnIA[A10L, D14K], ImI and LvIA [34,35,36,37,38,39,40], respectively, as well as *Lymnaea stagnalis* (Ls) AChBP bound by α-cobratoxin [41] and LsIA [27], respectively. 

**Figure 1:**
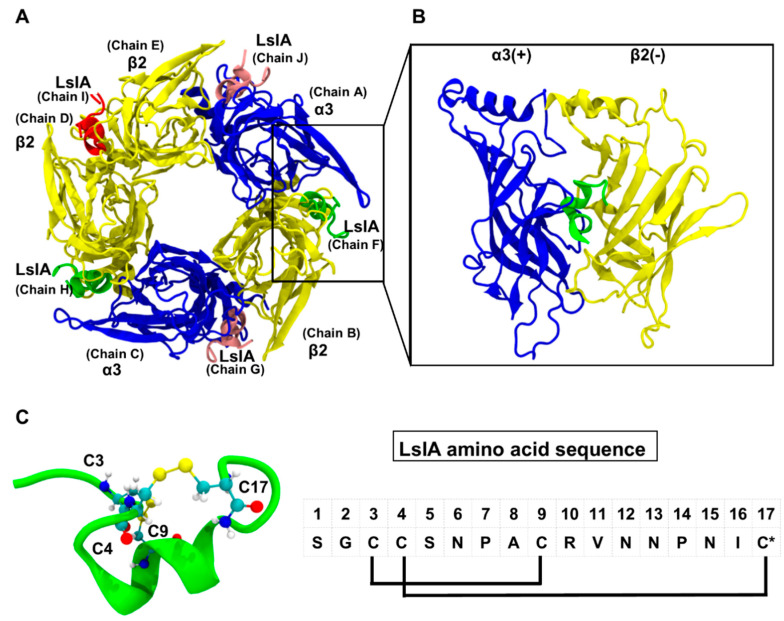
**Binding mode of α-conotoxin LsIA at rat α3β2 nAChR.** (**A**) Top view of LsIA anchored to the receptor. The five subunits of the α3β2 and the LsIAs anchoring to five interfaces of the nicotinic receptor are labelled with their nomenclature names and the chain numbers used to identify subunits in this work. The α3 and β2 subunits are represented by a blue and yellow colour, respectively, in NewCartoon form; and LsIAs bound at α3(+)β2(−), β2(+)α3(−) and β2(+)β2(−) sites are shown in green, pink and red colours, respectively. (**B**) Side view of LsIA binding at the α3(+)β2(−) interface. The homology model of the LsIA/α3β2 complex was constructed via homology modelling (Modeller9v6 [42], and was further refined via molecular dynamics (MD) simulation (GROMACS 4.6.5 [43]). A detailed description of the methods is provided in the Materials and Methods section. (**C**) The 3D structure of LsIA with 4 conserved cysteine residues shown in CPK form. The yellow bonds represent the two disulfide bonds of the toxin. The amino acid sequence of LsIA is shown on the right-hand side of the figure. The asterisk (*) denotes the C-terminal amidation.

In the present study, we employed MD simulations to predict the determinants for the differences in receptor interactions with amidated (LsIA) and carboxylated C-T (LsIA#) analogue of LsIA at α3(+)β2(−), β2(+)α3(−) and β2(+)β2(−) interfaces (see Figure 1), which may help to explain the differential inhibition of α3β2 by these two toxin isoforms. These results predict differences in the structures, motions and intra-molecular contacts between LsIA# and LsIA, as to elucidate the toxin-receptor interactions which are increased for LsIA# compared to LsIA at certain types of interfaces, which may thereby be proposed to be key determinants for the increased inhibitory activity of LsIA# at α3β2. The electrostatic property that affects the binding of LsIA# at α3β2 versus its native type (LsIA) was also explored via Adaptive Poisson–Boltzmann Solver (APBS). Furthermore, in addition to toxin-receptor contacts and their physical characteristics, we examined the structural influences exerted on the receptor by the two LsIA isoforms and employed a molecular network approach to compare the effects that each LsIA isoform may exert on contacts between adjacent nAChR subunits at α3(+)β2(−) and β2(+)α3(−) interfaces. The consideration of inter-subunit contacts to predict the potential bioactivity of α-conotoxins bound at specific nAChR interfaces is worthy of further investigation. 

## 2. Results

### 2.1. Stability and Flexibility of the LsIA-α3β2 Complexes

**Figure 2:**
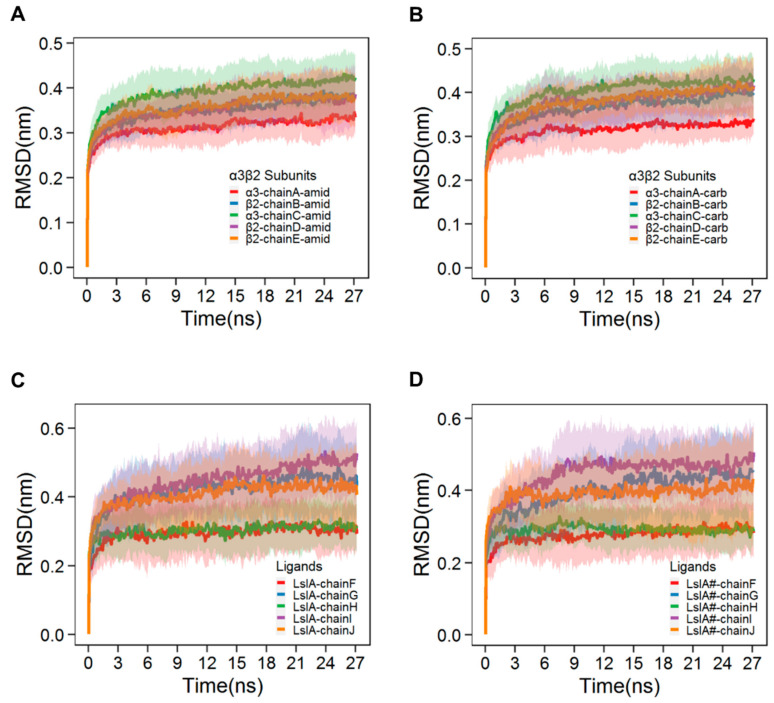
**Root-mean-square deviation (RMSD) (Mean ± SD) plots of LsIA and its carboxylated analogue (LsIA#) binding at α3β2 pentameric nicotinic acetylcholine receptors (nAChR).** (**A**) RMSD plots of LsIA bound α3β2. (**B**) RMSD plots of LsIA# bound α3β2. ‘Amid’ represents the amidated LsIA bound to α3β2 nAChR, whereas ‘Carb’ represents the carboxylated LsIA (LsIA#) bound α3β2 nAChR subunits. RMSD plots of (**C**) LsIA and (**D**) LsIA# upon anchored to the nAChR.

The root-mean-square deviation (RMSD) time series plots for the LsIA- and LsIA#-α3β2 complexes are shown in Figure 2A–D, each time series of which was taken as an average over 28 independent simulations. The fitting was performed on the Cα atoms of the entire receptor-ligand complex and RMSD values were calculated for the respective individual chains, discussed below. These plots demonstrate the stability of the backbone atoms of the protein complexes [44], all of which approach a plateau in their respective RMSD plots by approximately 15 ns. The patterns of the RMSD plots in the present study are qualitatively comparable to those of the RMSD results based on simulations of other nAChRs-toxins models [45,46,47]. Despite the similarity of the RMSD plots of the α3β2 nAChR subunits between LsIA and LsIA# bound forms, there are, however, some differences in the RMSD behavior observed for both of the ligands. LsIA and LsIA# show an asymmetric pattern in their RMSD plots, depending on the targeted α3β2 interfaces bound by the toxins, as shown by Figure 2C,D. The figures demonstrate two distinct types of RMSD values. Chain G (bound at the β2(+)α3(−) interface), chain I (β2(+)β2(−)) and chain J (β2(+)α3(−)) exhibited high values of RMSD (approaching 0.5 nm), whereas chain F (bound at α3(+)β2(−)) and H (α3(+)β2(−)) exhibited much lower RMSD values (approaching 0.3 nm). The RMSD results indicate that LsIA, when bound at interfaces involving β2(+) as the principal face, went through substantially greater structural drift from the initial homology model compared to those bound at the α3(+) interfaces. This asymmetry may be because the β2(+)α3(−), and β2(+)β2(−) interfaces are less preferable for the binding of LsIAs, compared with the α3(+)β2(−) interfaces. 

To obtain further insights into the regions of LsIA responsible for the interface-specific differences in RMSD as described above, absolute root-mean-square fluctuation (RMSF) plots were obtained for the LsIA- (Figure S1A,C) and LsIA#-α3β2 complexes (Figure S1B,D). To confirm that the structural and dynamical properties of the LsIA-nAChR complexes were reasonably converged within the simulation time frame, we plotted the sum of squared differences (SSD) at 3, 6, 9, 12, 15, 18, 21, 24 and 27 ns RMSFs for the average of the trajectories (Figure S2), as well as individual trajectories (not shown). These plots indicate that 21 ns is sufficient for convergence, at least within the timescale of the present simulations (Figure S2).

In the RMSF results for LsIA and LsIA# (Figure S1C,D), a high fluctuation in the N-terminus (N-T) of LsIA was observed. For both LsIA and LsIA#, the values of the RMSF followed the same pattern as the RMSD, with chains G, I and J (binding at β2(+) interfaces) exhibiting distinctly higher RMSF compared to chains F and H (binding at α3(+) interfaces), particularly in the N-T and N15 regions. Thus, the N-T of both LsIA and LsIA# appear to undergo far greater flexibility at β2(+)α3(−) compared to those bound at α3(+)β2(−) interfaces. The differences, mainly regarding the N-T of LsIAs, may suggest important variations in toxin-receptor interactions, depending on the interface arrangement.

**Figure 3:**
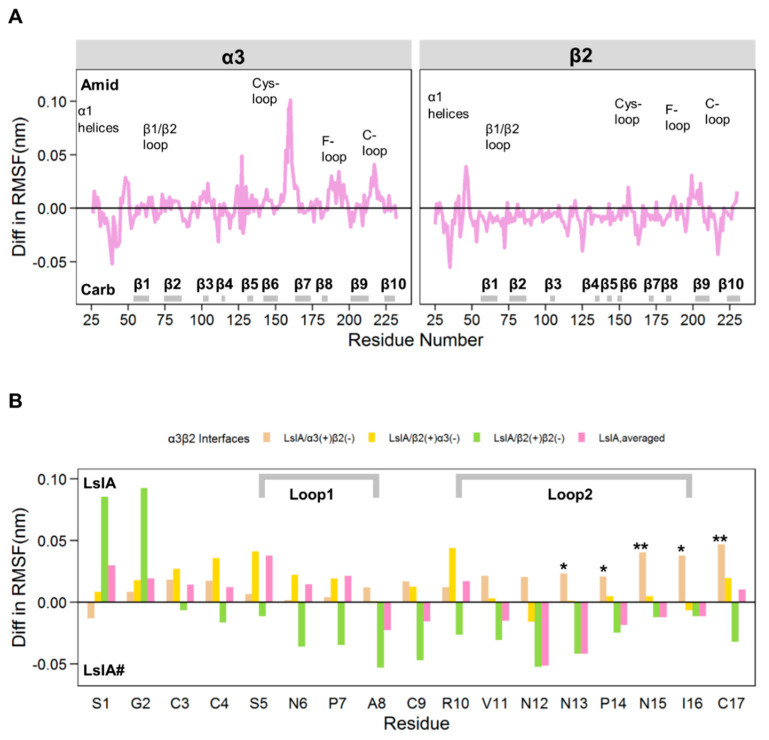
**Differences between RMSF plots of LsIA and LsIA# binding at α3β2 nAChR.** (**A**) Averaged diff in RMSF plot of α3β2 subunits bound by LsIA. The ’Amid’ label on the positive side of the y-axis represents the LsIA bound subunits to show higher flexibility versus the LsIA# bound form, whereas the ’Carb’ label on the negative side of the axis shows that the LsIA# bound form gains higher flexibility over the LsIA bound subunit. (**B**) Diff in RMSF for ligands anchoring to the nicotinic receptor interfaces. The differences of RMSF between LsIA and LsIA# (The RMSF value of LsIA# was subtracted by that of LsIA) are demonstrated, with different bar colours for the LsIA binding at different interfaces. The diff in RMSF of LsIA binding at α3(+)β2(−), β2(+)α3(−), and β2(+)β2(−) interfaces are shown with beige, yellow and green, colours respectively, whilst the averaged diff in RMSF of LsIA is shown in pink colour. The statistical significance of changes in the RMSF of α3 and β2 subunits bound by LsIA/LsIA# and the individual residues of LsIA/LsIA# (binding at different interfaces), was calculated over 28 individual seeds (* *p* < 0.05; ** *p* < 0.001).

We also compared the differences in RMSF results between LsIA- and LsIA#-α3β2 complexes, by subtracting the RMSF values of LsIA# bound α3β2 nAChR from those of the LsIA bound complex (Figure 3A). Positive values represent enhanced flexibility of the backbone of the LsIA bound complex, whereas lower flexibility (relative to LsIA#) is demonstrated by negative values. Only residues that show differences in RMSF values greater than 0.5 (Å) were included, as this value has been used as a cut-off point in previous MD simulation studies comparing similar proteins in different states [48,49]. 

For α3β2 (Figure 3A), there is an increased RMSF value in some regions, including Cys-loop and C-loop of the LsIA-bound α3 subunit versus the LsIA# complex, illustrating the enhanced flexibility in this region. Greater C-loop flexibility may infer a lower capacity for LsIA to lock the ligand-binding site in an open-loop, inhibited conformation, or may be indicative of weaker receptor-toxin interactions for LsIA leading to higher flexibility. In contrast, for the β2 subunit, the opposite result was observed, showing that the rigidity of these regions is higher for the LsIA bound subunit compared to the LsIA#. In contrast to the observation regarding the C-loop of the α3 subunit, the enhanced rigidity of the β2 subunit bound by LsIA, may suggest that LsIA has less effect on suppressing Cys-loop and C-loop movements at α3(+) rather than β2(+) interfaces. However, the increased flexibility of residues in the Cys-loop and C-loop of the α3 subunit was not significant at the 5% level, except for the two residues, α3-Y215 (P-value 0.031) and α3-C217 (*p*-value 0.016), which showed statistically significant differences from the α3 subunit of α3β2 nAChR bound by LsIA#. Further, the α3-Y215 residue played an important role in ACh binding at α3(+)β2(−) interfaces, by forming aromatic interactions with the neurotransmitter [20]. 

For LsIA (Figure 3B), the differences in RMSF values are shown for the different interfaces of the α3β2 nAChR bound by the toxins. At α3(+)β2(−) interfaces, LsIA# was less flexible than LsIA at most positions, especially for the residues, N13, P14, N15, I16 of loop 2 and the C-T (beige bars, Figure 3B). This suggests that at α3(+)β2(−), LsIA# may form a higher number of intermolecular contacts with the receptor, or may form internal intra-molecular contacts, which can result in its significantly greater rigidity. At the β2(+)α3(−) interfaces, LsIA# was, however, less flexible than LsIA in only loop 1, whereas LsIA# exhibited similar flexibility to LsIA in loop 2 (yellow bars, Figure 3B), in contrast to the observed RMSF behavior for α3(+)β2(−) above. Finally, at the β2(+)β2(−) interface, a different RMSF behavior was observed; aside from LsIA-G1 and S2, LsIA# exhibited higher flexibility for both loops 1 and 2 compared to LsIA (green bars, Figure 3B). Thus, at both mixed subunit interfaces, the structure of LsIA# was generally more rigid than LsIA, but at β2(+)β2(−), LsIA# was only more rigid at residues 1 and 2, but more flexible elsewhere in both loops 1 and 2. Therefore, we propose that the enhanced rigidity of LsIA# compared to LsIA at α3(+)β2(−) interfaces is qualitatively consistent with the expectation that LsIA# should have greater toxin-receptor contacts than LsIA (and therefore less mobile), given the known higher potency of LsIA# at α3β2. There is also an indication that at the β2(+)α3(−) interfaces, LsIA# might be marginally more rigid than LsIA, although caution must be exercised in this interpretation, due to lack of statistical significance at the 5% level for the results at this type of interfaces. It is plausible that considering both these mixed interfaces may help elucidate the toxin’s differential potency. However, the same cannot be said for β2(+)β2(−), for which there was generally higher flexibility for LsIA#, suggesting less toxin-receptor contacts. Thus, the consideration of β2(+)β2(−) is less likely to have a predictive value, and in subsequent sections, we focused our discussion mainly on α3(+)β2(−) and β2(+)α3(−) interfaces, with the majority of the β2(+)β2(−) results provided in Supplementary Materials. To elucidate the determinants affecting the possible differences at α3β2 interfaces’ binding potency by LsIA and its analogue, we further investigated the basis for the distinctions in RMSF behavior between LsIA and LsIA# at the α3(+)β2(−) and β2(+)α3(−) interfaces, by examining specific intra- and inter-molecular contacts between LsIA and the α3β2 receptor discussed below.

### 2.2. LsIA and LsIA# Toxin-Receptor Contacts

**Figure 4:**
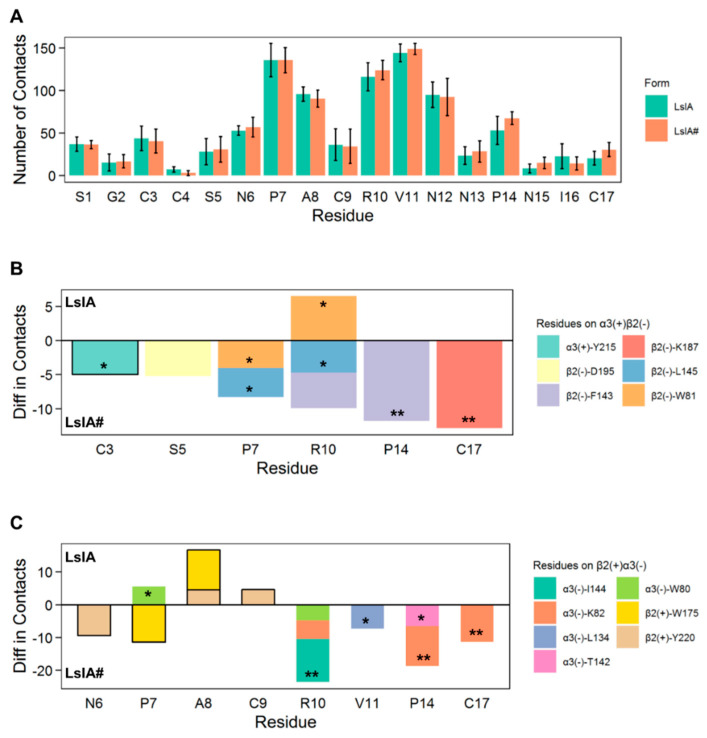
**Summary of the number of contacts in LsIA and LsIA# bound forms.** (**A**) A total number of receptor interactions by LsIA and LsIA# (Mean ± SD). The mean values were calculated over five α3β2 interfaces, and the error bars represent the standard deviation (SD). The significant changes of pairwise contacts for LsIA anchored to α3(+)β2(−) interfaces (**B**) and β2(+)α3(−) interfaces (**C**) of α3β2 nAChR by C-terminal carboxylation. The residues on the principal (+) face of binding interfaces of α3β2 are shown with a black line border. The statistical significance of the difference between the number of pairwise interactions was calculated over 28 individual seeds (* *p* < 0.05; ** *p* < 0.001).

The total number of contacts for each residue of LsIA formed with α3β2 is depicted in Figure 4A, whereas Figure 4B,C and Figure S3 show the important pairwise interactions influencing receptor interactions by the LsIAs anchored to the α3(+)β2(−) (Figure 4B), β2(+)α3(−) (Figure 4C) and β2(+)β2(−) (Figure S3) interfaces. 

Overall, the LsIA# with α3β2 nAChR complex had higher inter-residue contacts between the ligand and the receptor versus the LsIA bound complex, as can be seen in Figure 4A. The total number of receptor interactions made by LsIA was mainly contributed by P7 on loop 1, and with R10 and V11 on loop 2 for both LsIA and LsIA#. The higher toxin-receptor contacts may partially explain the higher rigidity of LsIA# at both α3(+)β2(−) and β2(+)α3(−) interfaces, compared with the LsIA bound complex. 

In terms of the specific determinants affecting the total receptor interactions by LsIAs, pairwise interactions between the receptor and the LsIAs were further investigated via *gmx mindist* with cutoff 4.5 Å. To make the differences in per-residue contacts between LsIA and LsIA# at the α3(+)β2(−) interfaces clear, the number of individual pairwise contacts of LsIA# was subtracted by that of LsIA. Thus, a negative value represents relatively higher LsIA# contacts, whereas the positive value shows higher contacts for LsIA with the corresponding residues on α3β2 nAChR, which is indicated within the bars for each LsIA residue. The absolute value of 4 for the differences in the number of contacts for coupled residues was selected as a threshold for including the potentially important pairwise contacts affecting the receptor interactions. Additionally, for ease of interpretation, pairwise interactions were excluded if the original number of contacts, between LsIA and LsIA# bound complexes, was less than 15, indicating a low level of contacts formed by the coupled residues. The statistical significance was evaluated for the selected pairwise interactions over 28 individual simulations. It is shown that P7, P14 and C17 formed the most significant variations in several contacts between LsIA and LsIA# binding at both α3(+)β2(−) and β2(+)α3(−) interfaces (Figure 4B,C), and fewer differences occurred at the β2(+)β2(−) interfaces (Figure S3). The interactions involving each of the above LsIA residues at the α3(+)β2(−) and β2(+)α3(−) interfaces are discussed in turn in the following sections.

### 2.3. Important Pairwise Interactions of LsIA with Residues at α3(+)β2(−) Interfaces

**Figure 5:**
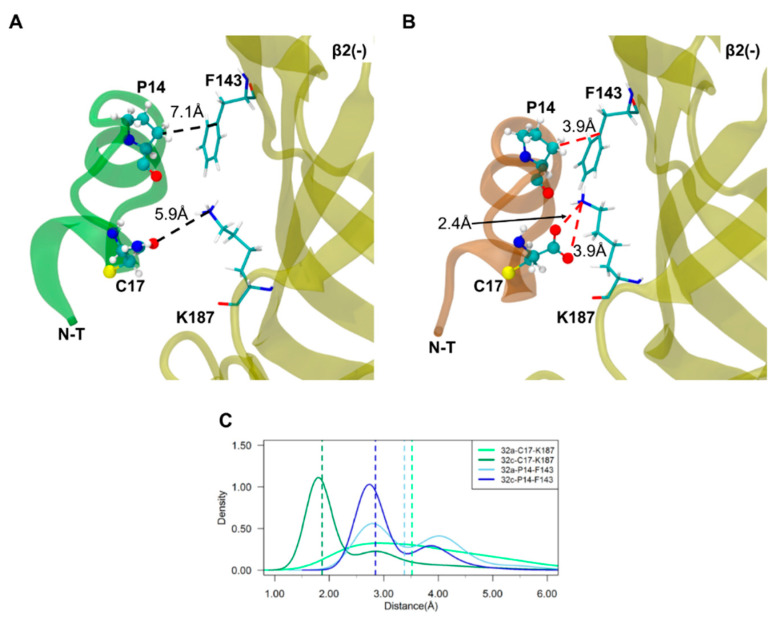
**Important interactions formed by LsIA-P14 and C17 with the receptor residues on the complementary (−) face at the α3(+)β2(−) interface**. Binding mode of LsIA (transparent green) (**A**) and LsIA# (transparent orange) (**B**) at the (−) face with the pairwise interactions which may substantially affect the binding affinity. The key residues involved in pairwise interactions on LsIAs are shown in CPK form, whereas the corresponding residues on the receptor are depicted with the Licorice form (a drawing format of graphic representation in visual molecular dynamics (VMD)). The red dashed line represents the contacts that are much stronger in this form of LsIA/α3β2 complex. Snapshots were chosen by visual inspection for illustrative purposes. (**C**) The distance (Å) between the coupled residues of bound forms. Moreover, *32a* represents the LsIA bound type, whereas *32c* denotes the LsIA# bound complex. The coloured dashed lines represent the median distance of the pairwise residue. The y-axis shows the probability density function of the distance between the coupled residues, which is involved in all the distance (Å) plots in this study.

**Figure 6:**
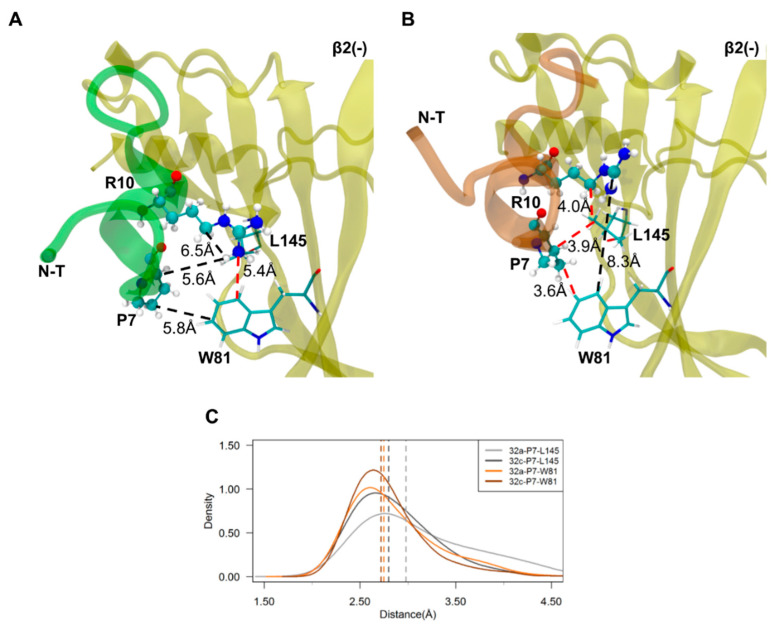
**Important interactions formed by P7 with the receptor residues on the complementary (−) face at α3(+)β2(−) interface.** Binding mode of LsIA (**A**) and LsIA# (**B**) at the β2(−) face with the pairwise interactions dramatically influences the binding affinity. (**C**) The distance (Å) between the coupled residues of bound forms that affect the changes in α3(+)β2(−) interface interactions by the toxin.

The increased contacts between LsIA#-C17 and β2(−)-K187 likely exerted the greatest impact on improved binding affinity of LsIA# versus LsIA at α3(+)β2(−) interfaces, together with P7 and P14 forming hydrophobic interactions with corresponding aromatic and hydrophobic residues, F143, L145 and W81, on the β2 subunit. The dominant enhanced contacts occurred between LsIA#-C17 and β2(−)-K187 via salt-bridge/hydrogen bonds interactions, which were absent in the LsIA/α3β2 complex (Figure 5 and Figure S4). Additionally, the proximity between P7 and β2(−)-W81 and β2(−)-L145 and the enhanced pairwise contacts between P14 and β2(−)-F143 (Figure 5 and Figure 6) contributed to the augmented hydrophobic interactions in LsIA# versus the LsIA bound form. 

Furthermore, the salt-bridge interaction between C17 and R10 of LsIA# (Figure S5) directly facilitated the LsIA#-R10 contact with β2(−)-L145, via forming van der Waals interactions (Figure 6), by placing the toxin in a favorable conformation for such interaction. Although the interactions formed by LsIA#-R10 and β2(−)-W81 were reduced against the LsIA bound form, the distance between the coupled residues was relatively large in the latter complex. Thus, the carboxylation of LsIA probably causes a switch in the favorable interaction of R10 with β2(−)-W81 (for LsIA) to β2(−)-L145 (for LsIA#). Nevertheless, as the van der Waals interaction between R10 and β2(−)-L145 was relatively weak, this “switch” may only result in a slightly overall increased number of contacts between the key residue R10 and the receptor at the α3(+)β2(−) interfaces by LsIA#. Consequently, the ability to form a salt bridge/hydrogen bond between the C-T and β2(−)-K187 is likely to be a key factor explaining the higher affinity of LsIA# compared to that of the amidated form LsIA at α3(+)β2(−) interfaces. This interaction may also play a critical role in helping produce an overall higher number of contacts between α3β2 and LsIA# compared to LsIA bound form, by drawing the toxin closer to the (−) face of the receptor, indirectly stimulating the significantly augmented contacts at other residues, such as R10 and P14, as discussed above. 

However, contrary to the β2 subunit at α3(+)β2(−) interfaces, the changes in receptor contacts at α3(+), between LsIA and LsIA# bound interfaces were minor, apart from significantly enhanced contact between α3(+)-Y215 and LsIA#-C3 versus the LsIA bound form (Figure S6). Other interactions, such as N6-(+)Y22, P7-(+)W174 and G2-(+)Y215 only slightly affected the binding affinity of the toxin due to C-terminal carboxylation (Figure S7).

### 2.4. Important Pairwise Interactions of LsIA with Residues at β2(+)α3(−) Interfaces

**Figure 7:**
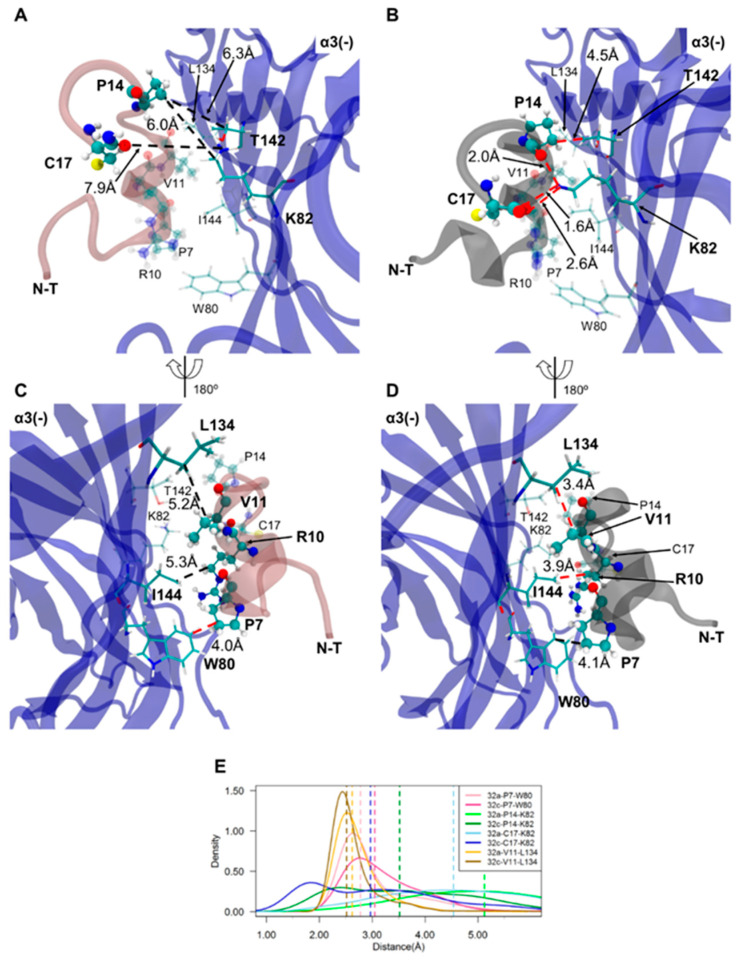
**Important interactions formed by LsIA-P7, R10, V11, P14 and C17 with the receptor residues on the complementary (−) face at β2(+)α3(−) interface.** Binding mode of LsIA (transparent pink) (rotated ~180° of the y-axis) (**A**,**C**) and LsIA# (transparent grey) (rotated ~ 180° of the y-axis) (**B**,**D**) at the (−) face with the pairwise interactions which may substantially affect the binding affinity. For better visualization, the unrelated residues in the figures are shown in transparent colour and labelled with smaller size font. (**E**) The distance (Å) between the coupled residues of bound forms.

Carboxylation of the C-T of LsIA also results in markedly enhanced pairwise interactions with residues on the α3(−) subunit of β2(+)α3(−) interfaces, such as and C17-(−)K82 and V11-(−)L134, via salt-bridge/hydrogen bond and hydrophobic interactions, respectively (Figure 4C and Figure 7). The increased number of receptor contacts for LsIA# was even more pronounced at β2(+)α3(−) than at the α3(+)β2(−) interfaces, except for the weakened contacts between LsIA#-P7 and α3(−)W80 versus the LsIA bound form. This is shown in Figure 4C, in which LsIA#, occupying the β2(+)α3(−) sites, had a greater relative number of contacts at the key residues demonstrated previously, such as P7, A8, R10, V11, P14 and C17 (with contact number differences of up to ~−30), compared to at α3(+)β2(−) (with contact number differences of only up to ~−15). This suggests that the carboxylation of C-T may cause a greater enhancement in binding affinity at β2(+)α3(−) compared to that at α3(+)β2(−) binding sites, such that the interactions at this interface may also be used to predict LsIA activity. Nevertheless, it should be noted that the changes in the number of contacts with statistical significance were commensurate with those at α3(+)β2(−), except for the additional substantially enhanced van der Waals contacts between R10 and I144 at β2(+)α3(−) bound by LsIA#. 

Similar to the α3(+)β2(−) interfaces, the C-terminal carboxylation may directly enhance the interactions established by LsIA#-C17 with a lysine residue at the (−) face, namely α3(−)-K82. Figure 4C and Figure 7 illustrate that LsIA# had greater contacts between the negatively charged C-T and α3(−)-K82 on β2-sheet of α3 subunit, which was also confirmed in the ligand interaction 2D diagram (Figure S4B,E). However, it appears that the proximity between α3(−)-K82 and LsIA#-C17 may not facilitate enhanced interactions by R10 and α3(−)-W80. At both β2(+)α3(−) and α3(+)β2(−) binding sites, the intramolecular interactions of R10-C17 in LsIA# may only interfere with the enhanced interactions between LsIA# and α3(−)-W80 as well as β2(−)-W81, via mainly favoring the binding of LsIA over LsIA# to the binding interfaces. Further, few variations of interactions occurred at β2(+) subunit (Figure S8), resembling the α3(+)β2(−) interfaces. 

In contrast, at β2(+)α3(−), R10 made more increased contacts with three residues for LsIA# relative to LsIA, namely, α3(−)-I144, K82 and W80 (Figure 4). Particularly, the α3(−)-I144 formed substantially enhanced contacts with LsIA#-R10, shown in Figure 7. However, at α3(+)β2(−), LsIA#-R10 had higher contacts only with two residues, β2(−)-L145 and β2(−)-F143. Apart from this, LsIA#-P14 made enhanced contacts with two receptor residues, α3(−)-K82 and T142 (Figure 7), relative to LsIA; this is in contrast to the α3(+)β2(−) interfaces, for which LsIA#-P14 established markedly enhanced contacts with a single residue, β2(−)-F143. 

### 2.5. Understanding Changes in Electrostatic Potential by C-terminal Carboxylation of LsIA at α3(+)β2(−) and β2(−)α3(+) Interfaces

In order to understand the effects of C-terminal carboxylation on the electrostatic properties of LsIA and α3β2 nAChR, which may shed further light on the differences in LsIA and LsIA# activities, we conducted an adaptive Poisson–Boltzmann solver (APBS) [50] analysis on LsIAs binding at α3(+)β2(−) and β2(+)α3(−) via PyMOL APBS plugin [51] (APBS Tools 2.1). PyMOL with an APBS plugin facilitates the visualization of electrostatic potential 3D surface molecules after calculation [52]. The Protein Data Bank format file of molecules was pre-processed via pdb2pqr [53] software for assigning parameters of the atoms, such as radius and charge, from various force fields [54]. 

**Figure 8:**
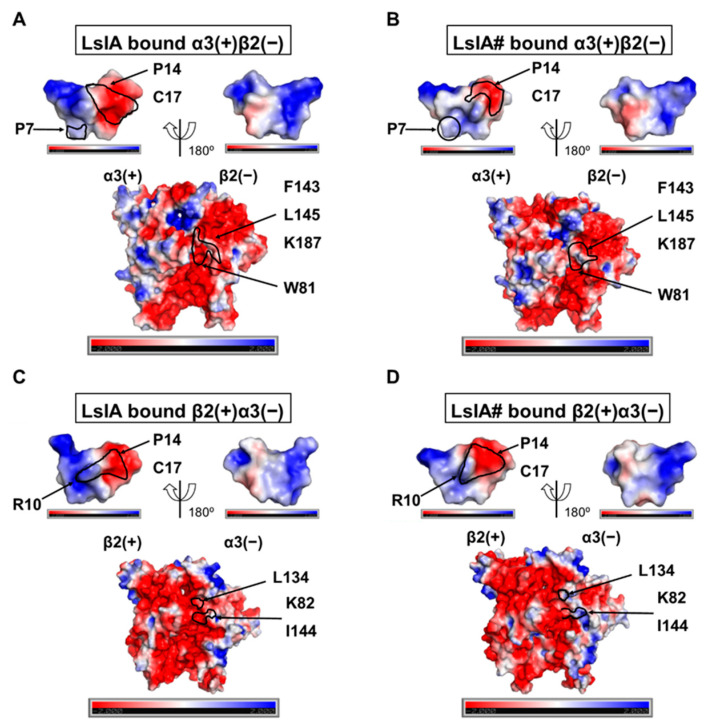
**Electrostatic potential energy on the surface of the α3β2 interfaces bound by LsIA/LsIA# shown in PyMOL [56].** (**A**,**C**) LsIA binds at α3(+)β2(−) and β2(+)α3(−) interfaces, respectively. (**B**,**D**) LsIA# binds at α3(+)β2(−) and β2(+)α3(−) interfaces of α3β2 nAChR. The electrostatic potential is within a range from −2 kT/e (red) to +2 kT/e (blue). The white color represents the neutral (0 kT/e)electrostatic potential.

The results demonstrated that the surface of C-terminal residues, C17 and P14, of LsIA obtain a larger negatively charged surface versus that of LsIA# (Figure 8A). Additionally, the pocket formed by β2(−)-F143, L145 and K187 on the β2 subunit of α3(+)β2(−) interfaces bound by LsIA were predominantly negatively charged. However, a relatively neutral/positively charged cavity formed by the corresponding residues on LsIA# bound interfaces was identified (Figure 8B). Therefore, electrostatic repulsion between LsIA and α3(+)β2(−) interfaces may substantially intervene in the α3β2 nAChR binding affinity versus the LsIA# bound form. For β2(+)α3(−), the surface of the binding cavity around α3(−)-L134 and K82 was dominated by neutral/positive charge when bound by LsIA# versus the LsIA anchoring form (Figure 8C,D), which resembled the α3(+)β2(−) binding site. We also observed an electrostatic interaction between α3(−)-K82 with LsIA#-R10 (Figure 8D),which may confer a stronger repulsion between them. This type of charge-charge interactions was also determined in previous studies, such as the interactions between α-conotoxin ImI-R11 and R148 and H146 on LBD of Ls-AChBP [55], as well as LsIA-R10-K57 on the β4(−) subunit of α3β4 nAChR [27]. Both of the electrostatic repulsions interactions are associated with the low binding affinity of ImI and LsIA at these nAChRs, compared to other nAChR subtypes, such as α7 and α3β2 nAChRs. 

### 2.6. Inter-subunit Contacts at the α3(+)β2(−) and β2(+)α3(−) Interfaces

To determine the effects of inter-subunit contacts between residues on the binding interfaces, we employed a network interaction analysis implemented in the software Cytoscape [57]. Differences in inter-subunit contacts help reveal changes in the relative orientations of the subunits at toxin-bound interfaces, which may be related to the capacity of the toxins to inhibit nAChR function [36,58,59,60]. 

**Figure 9:**
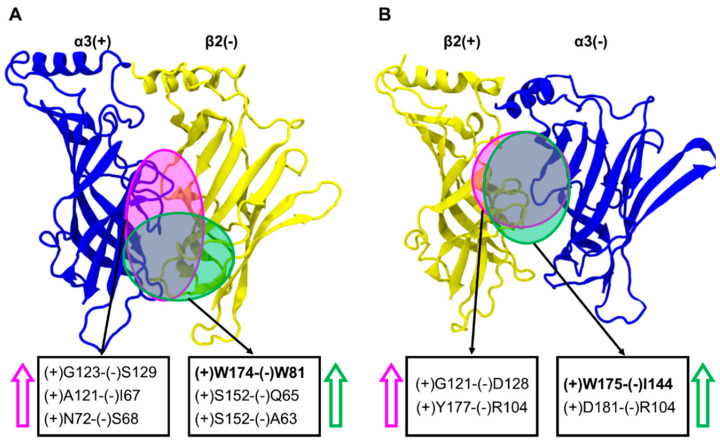
**Inter-subunit interactions between the principal and the accessory face of α3β2 nAChR interfaces bound by LsIA.** Contacts at α3(+)β2(−) (**A**) and β2(+)α3(−) (**B**) interfaces bound by LsIA and LsIA#. The region where differences in interactions occur due to anchoring of LsIA and LsIA# is shown with green and pink background colour, respectively. The pairwise interactions showing significant divergence in both forms are shown in bold form.

At the α3(+)β2(−) interfaces, α3(+)-S152 (on the Cys-loop) formed close hydrogen bond interactions with β2(−)-Q65 and β2(+)-A63, when bound with LsIA. These interactions were disrupted when LsIA# is bound, which is associated with an increased distance between the interfaces in the lower region (Figure 9A). In contrast, the van der Waals interactions formed by α3(+)-G123 and β2(−)-S129 were increased for LsIA# relative to LsIA, and is associated with a reduction in the distances between the interfaces in the middle part of the LsIA#-bound interfaces.

There was also a relative increase in distance between α3(+)-W174 and β2(−)-W81 for LsIA#-bound α3(+)β2(−) interfaces compared to LsIA. This corresponds to a shorter distance between these residues for LsIA-bound interfaces. The closeness between α3(+)W174 (on the β7/β8 loop) and β2(−)W81 (which lies on the β2 strand) when LsIA bound (Figure 9A and Figure S10), may have implications for how LsIA interacts with α3β2 interfaces. In particular, these contacts might lead to an upward shifting of (−)W81, which benefits the enhanced interactions between β2(−)W81 and LsIA-R10, and yet indirectly affect the changes in contacts in other important pairwise interactions. For example, LsIA-P7 and P14 formed reduced hydrophobic interactions with β2(−)-W81, L145 and F143 (on the β5 strand), associated with minor decreased contacts between the LsIA-R10 and β2(−)-L145, versus the LsIA# bound complex. 

Overall, at α3(+)β2(−) interfaces, LsIA# binding caused greater separation between the α3(+) and β2(−) subunits compared to LsIA at specific regions, including α3(+)S152 on the Cys-loop with β2 (−)Q65 and (−)A63, respectively, and α3(+)-W174 with β2(−)-W81, while reducing the separation at (+)G123 and (−)S129.

At the β2(+)α3(−) interfaces, we identified relatively strong hydrophobic interactions between β2(+)-W175 (also on the β7/β8 loop) and α3(−)-I144 (on β5 strand) for the LsIA#-bound form (Figure 9B and Figure S10), which may influence the differentiation between LsIA and LsIA# bound complex on β2(+)α3(−) interfaces, demonstrating greater contacts for the LsIA# and residues on the β7/β8 loop and (−)I144 by R10. In summary, LsIA# binding caused greater contacts between the subunits, drawing them closer, especially via the β2(+)-W175 and α3(−)-I144 residues. This is consistent with the contact difference plots (Figure 4), showing that at the β2(+)α3(−) interfaces, the binding of LsIA# resulted in a much greater increase in contacts relative to LsIA, when compared to that at the α3(+)β2(−). Part of this may be because LsIA# less effectively draws together the β2(+) and α3(−) subunits, which favors the interactions with the toxin relative to LsIA. 

## 3. Discussion

### 3.1. Different Combinations of the Interfaces in Rat α3β2 may Affect the Distinct Stability and Flexibility of the Receptor and LsIA

Simulations revealed differences in the overall dynamics and structure of the toxin-receptor complex when bound with either LsIA# or LsIA. In addition, there are also variations at the α3(+)β2(−) compared to β2(+)α3(−) interfaces. 

The examination of the RMSD shows interface-dependent differences. For the ligands (LsIA and LsIA#), a distinct pattern of the toxin RMSD values is observed at β2(+)α3(−) (chains G and J) and β2(+)β2(−) (chain I) interfaces, different from those binding at α3(+)β2(−) interfaces (chains F and H), for both LsIA and LsIA# bound complexes (Figure 2C,D). Although a negative charge at the C-terminal carboxylate of LsIA# may affect the conformation of the ligand as well, this asymmetry pattern arising at both LslA and LsIA# RMSD plots may clarify doubt about the conformational changes induced by C-terminal carboxylation. These differences in RMSD suggest that both LsIA and LsIA# may adopt different conformations at the α3(+)β2(−) versus β2(+)α3(−) and β2(+)β2(−) interfaces, particularly at the N-T of the ligand for both LsIA and LsIA#. Thus, these results may highlight the potential importance of nAChR interfaces in influencing the preferred conformations of bound conotoxins.

To explore the molecular basis of the differentiation between LsIA# and LsIA, as well as between the interfaces, the differences in RMSF value are also compared in this study, as shown in Figure 3A,B, indicating the different flexibility of the α3β2, and the toxins, for the LsIA and LsIA#-α3β2 complexes. The Cys-loop and C-loop of LsIA bound α3 subunit in the LsIA bound complex showed higher flexibility compared to the LsIA# bound subunit (Figure 3A). This appears consistent with the reduced rigidity of Cys-loop observed in several computational studies on α7 nAChR bound by antagonists versus the apo type [45,46]. However, to avoid the over-interpretation of the RMSF data, we also calculated the statistical significance of the RMSF differences between LsIA and LsIA# bound forms. As a result, only two residues, Y215 and C217 on the C-loop of α3 subunit, showed significantly enhanced rigidity upon binding by LsIA#.

For LsIA, the averaged differences in RMSF resemble those in LsIA bound at the human α7 nAChR, illustrating a more rigid structure of the loop 2 in the ligand, and relatively flexible backbone characterizing the loop 1 for LsIA# compared to LsIA. At both mixed subunit, α3(+)β2(−) and β2(+)α3(−) interfaces, the structure of the loop 2 of LsIA# is generally more rigid than LsIA. However, this observation (increased rigidity of loop 2 of LsIA#) is only critical at α3(+)β2(−) binding sites. The remarkably enhanced rigidity of LsIA# compared to LsIA at α3(+)β2(−) interfaces is qualitatively consistent with the expectation that LsIA# should have greater toxin-receptor contacts (and therefore less mobile) than LsIA does, given the known higher potency of LsIA# at α3β2. It is plausible that both of the mixed interfaces may need to be studied, particularly for the LsIA/α3(+)β2(−) interfaces, which show significant rigidity differences between the LsIA and LsIA# at loop 2 and C-T. We explained the toxin’s differential potency in regard to their interactions with α3β2 in the following sections. 

### 3.2. C-terminal Carboxylation of LsIA Marginally Enhanced Overall α3β2 nAChR Contacts Versus LsIA

Overall, the total number of contacts formed by the LsIA# with the α3β2 nAChR marginally surpasses that of the LsIA (Figure 4A), which is consistent with the experimental study, concluding that the potency of LsIA at α3β2 is enhanced three-fold via the C-terminal carboxylation of the LsIA [9], as mentioned previously. In particular, LsIA#-C17 and P14 are two key residues which were identified to form remarkably greater contacts with the receptor compared to LsIA. The enhanced contacts of the LsIA# may be induced by the potential intra-molecular salt bridges or hydrogen bonds established by the C-T (C17) and the R10 (Figure S5), which enhanced the rigidity of the residues in loop2, namely V11, N13, P14, N15, I16 and C-T, rather than R10. This increased rigidity was also suggested in a previous experimental study using an electrophysiological recording approach and predicted in our computational study on LsIA/α7 nAChR systems [9,61]. Therefore, the C-terminal carboxylation not only directly results in the enhanced receptor interactions by the C-T, but also improves the receptor interactions by P14 of the LsIA#. 

However, the relatively reduced receptor contacts at C3, A8, C9, N12 and I16 may partly offset this slightly increased number of α3β2 interactions by LsIA# over the LsIA. Furthermore, among all the residues of LsIA/LsIA# strongly interacting with α3β2 nAChR, C3, P7, R10, N12 and P14 show larger variations in terms of the number of receptor contacts over five binding interfaces. This may suggest that the interactions by the LsIA/LsIA# with an individual type of α3β2 interfaces, namely α3(+)β2(−), β2(+)α3(−) and β2(+)β2(−), may differ from each other. Therefore, the identification of the statistically significant changes in receptor contacts by individual residues was implemented for the prediction of the key residues involved in enhanced α3β2 interactions by LsIA# versus LsIA, among all α3β2 binding interfaces. It is also of interest to explore if different α3β2 nAChR interfaces may affect the slightly reduced binding affinity of LsIA versus its C-terminal carboxylation form.

### 3.3. C-Terminal Carboxylation of LsIA Mainly Enhances the Contacts with Residues on the Complementary Subunit of both α3(+)β2(−) and β2(+)α3(−) Interfaces

The key important pairwise interactions include that formed by C17 and β2(−)-K187 via forming hydrogen bonds/salt bridge, which is reinforced in the LsIA# bound complex, together with a dramatically enhanced hydrophobic interaction between P14 and β2(−)-F143 on α3(+)β2(−) interfaces of α3β2 nAChR versus the LsIA-bound complex (Figure 5). The geometry of LsIA#-C17 confers strong hydrogen bonds or salt-bridges established by the carboxyl group of C17 with β2(−)-K187. In our previous study, similar hydrogen bonds between LsIA#-C17 and (−)Q79 of human α7 nAChR were determined [61]. Interestingly, salt bridges/hydrogen bonds formed by other α-conotoxins and the receptor are involved in anchoring the inhibitors to nAChRs. For example, β2(−)-K187 on α3(+)β2(−) interfaces of α3β2 nAChR was implicated in charge-charge interactions with E11 of the α-conotoxin MII [20], whereas human β2(−)-D171 (D196 as numbered from first Met) forms relatively strong contacts with G1 of α-conotoxin RegIIA via salt-bridge [62], when the identical aspartic acid residue is situated in position 195 of the rat β2 subunit. Further, the proximity between LsIA#-C3 and α3(+)-Y215 confers substantially enhanced hydrogen bond contacts by LsIA# with α3β2 nAChR (Figure S6). The (+)Y215 of α3 subunit has been suggested to be important for the binding of ACh to α3(+)β2(−) binding sites via aromatic interactions [20]. This is also the only residue on the (+) face at both α3(+)β2(−) and β2(+)α3(−) interfaces showing markedly different effects on the binding between LsIA and LsIA#. 

At the (−) subunit on the α3(+)β2(−) interfaces of α3β2 nAChR, other important differential interactions involve those formed by P7 and P14 with aromatic and hydrophobic residues, β2(−)-W81, F143 and L145, via hydrophobic interactions, which are significantly enhanced in the LsIA# bound complex versus the LsIA (Figure 5 and Figure 6). The hydrophobic interactions between residues on α-conotoxins and aromatic residues of nAChRs were also identified in a previous study. For instance, a hydrophobic interaction is formed between L15 of LtIA with F119 (F143 from first Met) on the rat β2 subunit of α3β2 nAChR [32]. This interaction favors the selectivity of LtIA to α3β2 nAChR versus the α3β4 subtype.

On the contrary, the contacts formed by LsIA#-R10 with β2(−)-W81 and L145 slightly affect the different receptor interactions between LsIA and LsIA#, by compensating the changes in contacts between each other. More importantly, the stacking interactions (cation-π and π−π interactions) by R10 were not observed for either LsIA or LsIA# bound interfaces. This finding differs from the previous studies, illustrating the important role of stacking contacts in α-conotoxins anchored to nAChRs. The stacking interactions formed by the antagonists with aromatic residues on nAChRs were determined in numerous previous studies on α-conotoxins and ACh binding at nAChRs [6,30,35]. The proximity of R7 of α-conotoxin ImI with the aromatic residue, namely Y195, W149 and Y92 on the (+) face of the human α7 nAChR, may form cation-π interactions for stabilizing the ligand and receptor complex [1,31]. Similar π−π interactions were found between α-conotoxin MII-H9 and H12 and (−)F119 at α3(+)β2(−) interface and the (−)W55 at β2(+)α3(−) interfaces of rat α3β2 nAChR by Sambasivarao and colleagues [20]. Other cation-π and π−π interactions were determined in the F9 of AuIB with W59 and K61 on β4(−) subunit of α3β4, and LsIA-R10 with (−)W77 of human α7 nAChR, respectively [36,61]. 

The residues on position 5 of α-conotoxins may contribute to the potency of toxins at nAChRs via establishing polar interactions with negatively charged residues. In this study, the persistent hydrogen bond interactions between S5 and β2(−)-D195 stabilized the toxins bound to α3β2 nAChR in both LsIA and LsIA# complexes (Figure S4A,D). It has been suggested that this aspartic acid on rat β2 subunit establishes salt-bridge interactions with the R5 of LtIA, associated with S168 [32], rendering the toxin with a shallow binding position versus other α-conotoxins targeting nAChRs. In addition, the aspartic acids in position 197 and 195 at the α3(+) subunit of rat α3β2 and Ac-AChBP, respectively, also play an important role in the increased sensitivity of TxIA[A10L] to the receptors via electrostatic interactions with R5 of the toxin [34]. Taken together, our results support the important role of the charged residues on the F-loop of the β2 subunit of α3β2 nAChR in selectivity by α-conotoxins. 

At β2(+)α3(−) interfaces, for LsIA#, a salt bridge/hydrogen bond interaction exists between α3(−)-K82 and C17, together with other major hydrophobic interactions, such as LsIA#-V11 and α3(−)-L134 (Figure 7 and Figure S4), for increasing the binding affinity by the toxin. Similarly to α3(+)β2(−) interfaces, the LsIA#-C17 formed a substantially enhanced interaction with α3(−)-K82 via electrostatic interactions (Figure 8). The hydrogen bond/salt bridge interaction exerts the most critical impact on enhancing the binding affinity of LsIA# at α3β2 nAChR, associated with another substantially increased pairwise contacts between LsIA-V11# and α3(−)-L134 via hydrophobic interactions. The α3(−)-L134 residue is also a homologous residue at position 107 of the human α7 subunit, in which the (−)L107 of α7 subunits forms hydrophobic interactions with ImI-W10 [31]. We also observed a proximal distance between LsIA#-R10 and α3(−)-I144 for forming van der Waals interactions versus the LsIA bound form, with a resemblance to the enhanced van der Waals interactions between LsIA#-R10 and β2(−)-L145 at α3(+)β2(−). LsIA# binding generally causes a higher association between the subunits at the Cys-loop regions compared to LsIA at α3(+)β2(−) (Figure 9). At β2(+)α3(−) interfaces, the binding of LsIA# binding causes slightly reinforced contacts between the subunits, drawing them closer together, except for the β2(+)-W175 and α3(−)-I144 residues. This is consistent with the contact difference plots (Figure 4), which show that at both interfaces, LsIA# shows a higher number of contacts with α3β2 compared to LsIA. This may, in part, be due to LsIA# being more able to effectively draw together the subunits. Additionally, α3(−)-I144 is also homologous with (−)L141 on α7 subunit of human α7 nAChR, which was determined to form an enhanced interaction with LsIA-R10 of LsIA versus the carboxylated analogue [61]. In summary, the hydrophobic residues, isoleucine and leucine, at this homologous position, may contribute to the enhanced interactions by LsIA# at α7 and α3β2 nAChRs versus the wild type. 

Nevertheless, the interactions between LsIA#-P7 and α3(−)-W80 are reduced, in contrast to the binding of LsIA via establishing hydrophobic interactions with the pyrrolidine ring of P7. Thus, the role of α3(−)-W80 and β2(−)-W81 putatively favors the binding of LsIA compared with LsIA# at both α3β2 interfaces via hydrophobic or van der Waals interactions, despite enhanced interactions between P7 and β2(−)-W81.

The electrostatic repulsion may undermine the contacts formed by LsIAs-R10 with aromatic residues at the β2(+)α3(−) interface via stacking interactions. The loss of aromatic/stacking interactions between LsIAs-R10 and corresponding aromatic residues probably bears a close resemblance to that on α3(+)β2(−) interfaces. Neither was a significant and strong change in such interactions by R10 observed. The closer distance between LsIA#-R10 and C17 via intramolecular salt bridge interaction may induce the electrostatic repulsion between LsIA-R10 and K82 (Figure 8), which forms dramatically enhanced interactions with LsIA#-C17. A previous study showed that the LsIA [R10F, N12L] facilitates the selectivity of LsIA binding at human α3β4 over α7, Ls-AChBP and Ac-AChBP [27]. Thus, further study may require the mutagenesis of LsIA#-R10, possibly substituted by hydrophobic residue, for increasing the binding affinity of toxins at α3β2. Other studies also suggested the important role of hydrophobic residues, such as alanine and leucine, at position 10 of toxins, for the augmented inhibition of nAChRs by PnIA, PnIB and PeIA [S9H,V10A], respectively. A10 of PnIA and L10 of PnIB are critical for the inhibitory effects by the peptides at α7, α3β4, and α3β4 nAChRs, respectively [34,63,64]. The replacement of alanine by leucine in position 10 of PnIA reduced the sensitivity to α3β2 [64]. Multiple amino acid substitutions, including position 10 of certain α-conotoxins, can also affect the inhibition of nAChR subtypes. PeIA [S9H,V10A], for example, enhanced the selectivity of the toxin to α3β2 and α6/α3β2β3 [65]. 

The findings in this computational study may provide data for further experimental study. The in vitro cells, such as *Xenopus laevis* oocytes [34], human embryonic kidney (HEK-293) cells [66] and SH-SY5Y cells [67], are widely applied in the functional characterization of nAChRs and their sensitivity to their potential antagonists (e.g. α-conotoxins and cocaine) [13,20,21,30,32,62,66,68,69], namely, α3β2, α3β4, α7, α7β2, and AChBP for the crystallization of the structure. Among these cell lines, the most commonly used cell is the *Xenopus* oocyte, which has been demonstrated to successfully express different α3β4 and α9α10 nAChR stoichiometries [16,18]. 

## 4. Materials and Methods

In the present study, the complex of α-conotoxin LsIA- and LsIA#-α3β2 nAChR was first established via homology modelling with an appropriate template. The MD simulation was subsequently applied for refining the comparative models and providing the trajectory, including the geometries, velocities and energy of the atoms [70] for further analysis.

### 4.1. Homology Modelling

The comparative model of LsIA- and LsIA#-α3β2 nAChR complexes were built according to the process described in our previous study [61]. Briefly, the sequence of LsIA bound to the extracellular domain (ECD) of rat α3 subunit (UniProtKB-P04757) and rat β2 subunit (UniProtKB-P12390) was aligned to that of α-conotoxin PnIA[A10K, D14K] bound to *Aplysia californica* acetylcholine binding protein (Ac-AChBP) (PDB ID: 2BR8) [35], with the known crystal structure. The structure with the best discrete optimized protein energy (DOPE) score was selected from 100 models built via Modeller9v6 [42]. The DOPE score is calculated based on the distance-dependent energy function applied in the homology modelling approach, Modeller, and is used to evaluate the homology models at the atomic level [71]. The lower the DOPE, the better the model. The top view of the homology model of the LsIA-bound α3β2 nAChR interfaces is shown in Figure 1A, whilst Figure 1B demonstrates the side view of the motif of LsIA anchored to α3(+)β2(−) interface. The strategy, in which the model was constructed for LsIA binding at five interfaces of α3β2 nAChR, was also widely employed in other studies on α-conotoxin bound heteropentameric nAChR complexes [20,27,72]. It should be noted that the commonly assumed order of (α3)_2_(β2)_3_ for the homology modelling of the nicotinic receptor is α3−β2−α3−β2−β2 [19,24]. Although there is a lack of studies of concatemeric constructs of α3β2 nAChR, classic concatemer studies of α4β2 by Carbone et al. [73] indicated that functional receptor assemblies do not involve triplets of the same subunit. Thus, the αβαββ assembly motif is assumed to constitute the functional form of (α3)_2_(β2)_3_ as well.

As in our previous study on LsIA-α7 complexes [23], we noted that a recent co-crystal structure is available of Ls-AChBP, bound to amidated α-conotoxin LsIA (PDB code: 5T90 [29]), which showed minor structural differences in AChBP compared to all other conotoxin-Ac-AChBP crystal structures to date, possibly due to species differences between Ls- and Ac-AChBP. The authors constructed homology models of LsIA bound to α7 nAChR or apo α7 nAChR based on the Ls-AChBP crystal structure, and comprehensively elaborated and tested key interactions identified in the model in many elegant experiments [27,74]. In both cases, minor structural differences between Ls- and Ac-AChBP, and the availability of co-crystal structures of 4/7-conotoxins bound to both species isoforms, allow the exploration of an alternative, possible conformation of LsIA-α3β2 complex based on Ac-AChBP, which shares a comparable (though somewhat lower) sequence similarity to rat α3 and β2 subunits. 

In addition, the recently revealed X-ray structure of human α4β2 nAChR co-crystallized with nicotine (PDB ID:5KXI) [75] putatively supports the homology modelling of (α4)_3_(β2)_2_ nAChR stoichiometry [76] and molecular docking studies on α4β2 nAChR [77,78,79] for the small molecules, such as nicotine. Additionally, this determined structure companied with the complex of Ac-AChBP co-crystallized with the PnIA variant, benefits the comparative modelling of (α3)_3_(β4)_2_ and (α3)_2_(β4)_3_ nAChRs bound by α-conotoxin AuIB [15]. However, the nicotine bound α4β2 nAChR (PDB ID: 5KXI) [75] was only used for the construction of (α3)_3_(β4)_2_/ribbon isomer of AuIB (linking cysteine residues I-IV, II-III) complex [15], whereas the globular AuIB (linking cysteine residues I-III, II-IV) anchoring to (α3)_2_(β4)_3_ was based on the same template (PDB ID: 2BR8) [35] as in the present study. 

### 4.2. Molecular Dynamics Simulation

#### 4.2.1. MD Simulations on LsIA-α3β2 Complexes

MD simulations were carried out on the complexes using GROMACS 4.6.5 [43] and the CHARMM27 force field. The refined protein complexes were analyzed by following the process described previously [61]. To improve conformational sampling, each LsIA-α3β2 complex was run using 28 independent simulations, each initiated using a different random seed, up to at least 27ns. Unless otherwise stated, all subsequent results are reported as averages over the independent trajectories. Both systems were solvated with TIP3P water models [80]. For the LsIA-α3β2 complex, 70 mM Cl^−^ and 110 mM Na^+^ were added, to achieve an approximately 0.15 M salt concentration, whereas for the LsIA#-α3β2 complex, 70 Cl^−^ and 115 Na^+^ ions were added. Simulation trajectory frames were recorded every 10 ps for data analysis. The inter-subunit contacts were visualized through Cytoscape [57], which was also performed in our previous study [23]. All structure graphics were generated via visual molecular dynamics (VMD) [81]. Other simulation conditions and parameters are the same as those applied in our previous study [55]. The cluster analyses were performed over the whole trajectories for both LsIA and LsIA# bound forms via GROMACS packages. The trajectory frame interval was extracted every 10 frames, therefore around 7634 frames were acquired for clustering. The cutoff for LsIA- and LsIA#-α3β2 complexes was set up as 0.38 Å. As a result, the number of identified clusters for LsIA and LsIA# bound forms was 8 and 10, respectively. The top 1 ranked conformations (top 1 cluster) represent 92.4% (LsIA) and 91.4% (LsIA#) for the overall structural population for each protein complex, and were selected for APBS analysis [50] in the PyMOL APBS plugin [51] (APBS Tools 2.1). 

#### 4.2.2. MD Simulations on LsIA Only

To compare the intramolecular interactions within the LsIA/LsIA# in solution with their nAChR-bound forms, simulations were deployed only on LsIA and LsIA#, respectively, for 300 ns each. Consistent with the simulations on the receptor-ligand complexes, the result showed a proximate distance between R10 and C17 of LsIA# (3.44 Å ± 1.4) versus that of the LsIA (3.38 Å ± 0.96). For achieving 0.15 M concentration, the LsIA and LsIA# were solvated with 3 mM Na^+^ and 5 mM Cl^−^, and 3 mM Na^+^ and 4 mM Cl^−^, respectively, as well as water molecule models (TIP3P). The other parameters for simulation in this step are consistent with those for LsIA-α3β2 complex simulations. 

## 5. Conclusions

In the present study, we built molecular models of α3β2 nAChR bound by LsIA and LsIA# for an investigation of the effects of the C-terminal carboxylation of LsIA on the receptor interactions versus the wild type LsIA. The findings may provide new insights into elucidating the effects of different α3β2 binding interfaces, mainly between α3(+)β2(−) and β2(+)α3(−) interfaces, on the interactions by the LsIA and the residues on α3β2 nAChR.

An examination of the RMSD plots of LsIA at the α3(+)β2(−), β2(+)α3(−) and β2(+)β2(−) interfaces suggests that the toxins adopt remarkably different conformations at the latter interface, possibly indicating that the β2(+)β2(−) is unsuited to facilitate LsIA binding and inhibition. Subsequent discussions were, therefore, largely focused on the mixed-subunit interfaces. At both α3(+)β2(−) and β2(+)α3(−) interfaces, the carboxylated C-T (C17) forms strong hydrogen bonds/salt bridges with lysines at position 187 (at α3(+)β2(−) interfaces) and 82 (at β2(+)α3(−) interfaces), which occur together with the significantly enhanced interactions by P14 and P7 with hydrophobic residues (L145, W81 and F143) on the (−) face of α3(+)β2(−) interfaces. In addition, homologous residues, leucine and isoleucine, at positions 145 and 144 of β2(−) and α3(−) subunits, respectively, may contribute to the enhanced binding affinity of LsIA# at both interfaces via forming van der Waals interactions with LsIA#-R10. However, at β2(+)α3(−) interfaces, only LsIA#-V11 was observed to establish markedly augmented hydrophobic contacts with α3(−)-L134, compromised by the reduced hydrophobic contacts between LsIA#-P7 and α3(−)-W80. On the (+) face, β2(+)-Y215 exhibits enhanced hydrogen bond contacts with C3 of LsIA# versus the LsIA bound form at α3(+)β2(−) binding sites. By contrast, the significantly reduced contacts, for LsIA#, between β2(+)-Y177 and N12 at β2(+)β2(−) interfaces, decreased the total number of receptor contacts by LsIA#.

Combined with the enhanced rigidity of the residues (from position 13 to 17) of LsIA# over LsIA, we propose that the significant changes due to C-terminal carboxylation may occur predominantly at α3(+)β2(−) and β2(+)α3(−) interfaces, rather than β2(+)β2(−) sites, via hydrophobic and salt-bridge/hydrogen bond interactions at complementary faces. 

The determinants predicted in this study may provide information for the improvement of selectivity and specificity of LsIA to the nAChRs. Experimental studies employing the mutagenesis or synthesis of chemically modified α-conotoxins may be conducted in the future to improve the effectiveness of mediating nicotinic receptor activity as molecular drugs for novel therapies with fewer side effects.

## Supplementary Materials

The following are available online at https://www.mdpi.com/1660-3397/18/7/349/s1. **Figure S1**: RMS Fluctuation (RMSF) plots of α-conotoxin LsIA/LsIA# binding at α3β2 nAChR; **Figure S2**: Convergence study via calculating the sum of squared differences (SSD) between 3, 6, 9, 15, 18, 21, 24 and 27 ns RMSFs with the 27 ns simulations; **Figure S3**: Significant changes of pairwise contacts for LsIA anchoring to β2(+)β2(−) interface of α3β2 nAChR; **Figure S4**: The α3β2 nAChR interface interactions with LsIA and LsIA#, respectively, according to the statistically significant variations of receptor contacts by C-terminal carboxylation; **Figure S5**: Intra-molecular contacts between R10 and C17 of LsIA# versus LsIA binding at α3(+)β2(−) interfaces; **Figure S6.** Interactions formed by LsIA-G2 and C3 with the receptor residues on the principal (+) face at α3(+)β2(−) interface of α3β2 nAChR; **Figure S7**: Interactions formed by LsIA-P7 and C9 with the receptor residues on the principal (+) face at α3(+)β2(−) interface; **Figure S8**: Interactions formed by LsIA-P7 and A8 with the receptor residues on the principal (+) face at β2(+)α3(−) interface of α3β2 nAChR; **Figure S9**: Important interactions formed by LsIA-C9 and N12 with the relative residues on β2(+)β2(−) interface of α3β2 nAChR; **Figure S10**: The distance of the inter-subunit interactions between the principal (+) and the accessory (−) face of α3β2 nAChR interfaces bound by LsIAs. The trajectory data is available at the Zenodo repository (DOI: 10.5281/zenodo.3908769).
