# Supplementary material for: Interactions of the α3β2 Nicotinic Acetylcholine Receptor Interfaces with α-Conotoxin LsIA and its Carboxylated C-terminus Analogue: Molecular Dynamics Simulations
Source: Mar Drugs. 2020 Jul 3;18(7):349. doi: 10.3390/md18070349 (PMC7401271; doi:10.3390/md18070349)
Supplement: Supplementary file 1 [file marinedrugs-18-00349-s001.pdf]

Supplementary Materials:

Interactions of the $\alpha3\beta2$ Nicotinic Acetylcholine Receptor Interfaces with α-Conotoxin LsIA and its Carboxylated C-terminus Analogue: Molecular Dynamics Simulations

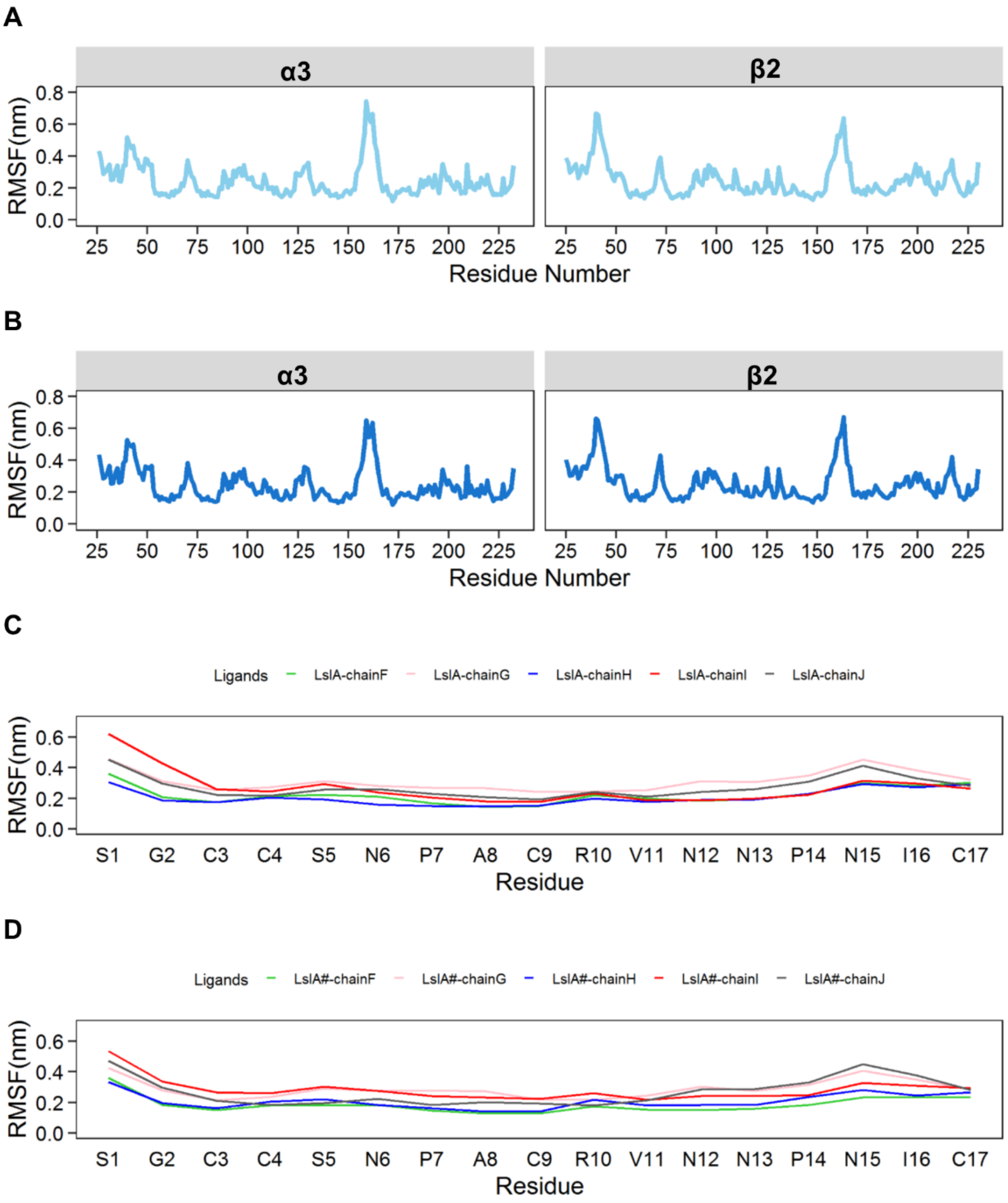

Figure S1. RMS Fluctuation (RMSF) plots of α-conotoxin LsIA/LsIA# binding at $\alpha3\beta2$ nAChR. RMSF plots of LsIA **(A)** and LsIA# **(B)** bound forms. RMSF plots of LsIA **(C)** and LsIA# **(D)** upon anchoring to the nicotinic acetylcholine receptor.

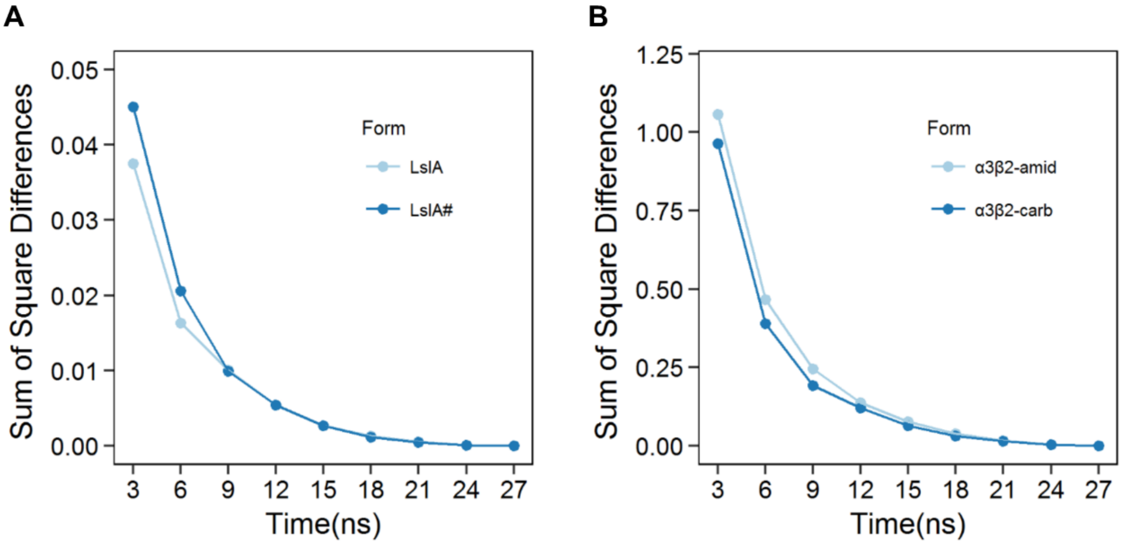

Figure S2. Convergence study via calculating the sum of squared differences (SSD) between 3, 6, 9, 15, 18, 21, 24 and 27 ns RMSFs with the 27 ns simulations. SSD of **(A)** LsIAs and **(B)** $\alpha3\beta2$ nAChR bound by LsIA and LsIA#. The higher SSD values indicate the greater overall difference from the 27 ns RMSF results. The figures show that there is an exponential decay in the difference in RMSF with increasing blocks of time, with the difference approaching zero from the 21 ns, which shows the RMSF from 21 ns is relatively similar to that of the 27 ns.

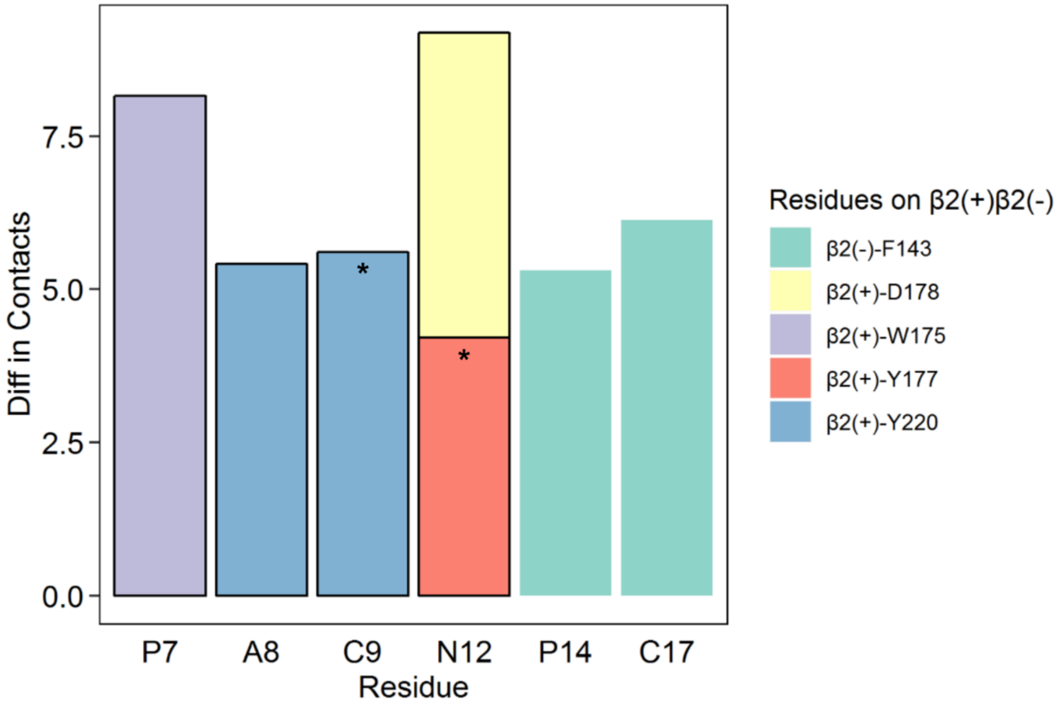

Figure S3. Significant changes of pairwise contacts for LsIA anchoring to β2(+)β2(–) interface of α3β2 nAChR. The residues on principal (+) face are labelled with a black line border. The statistical significance of the difference between the number of pairwise interactions was calculated over 28 individual seeds (* $P < 0.05$).

LsIA / α3β2

A

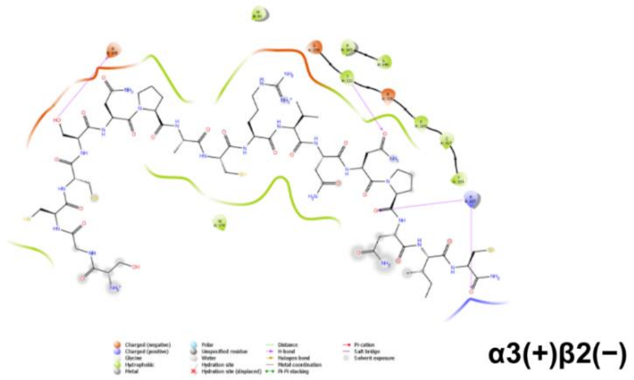

B

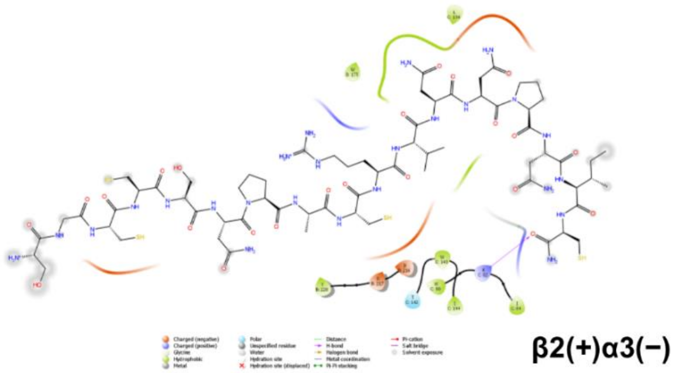

C

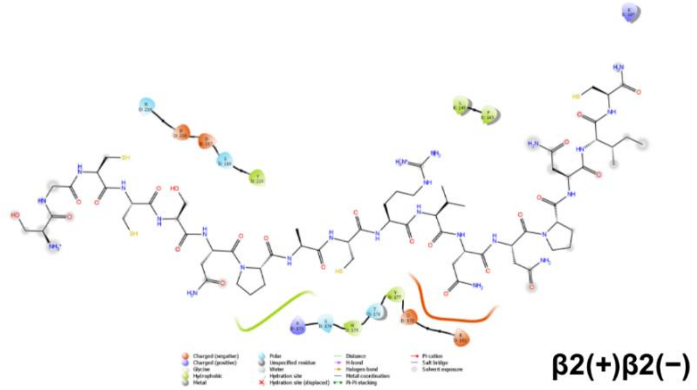

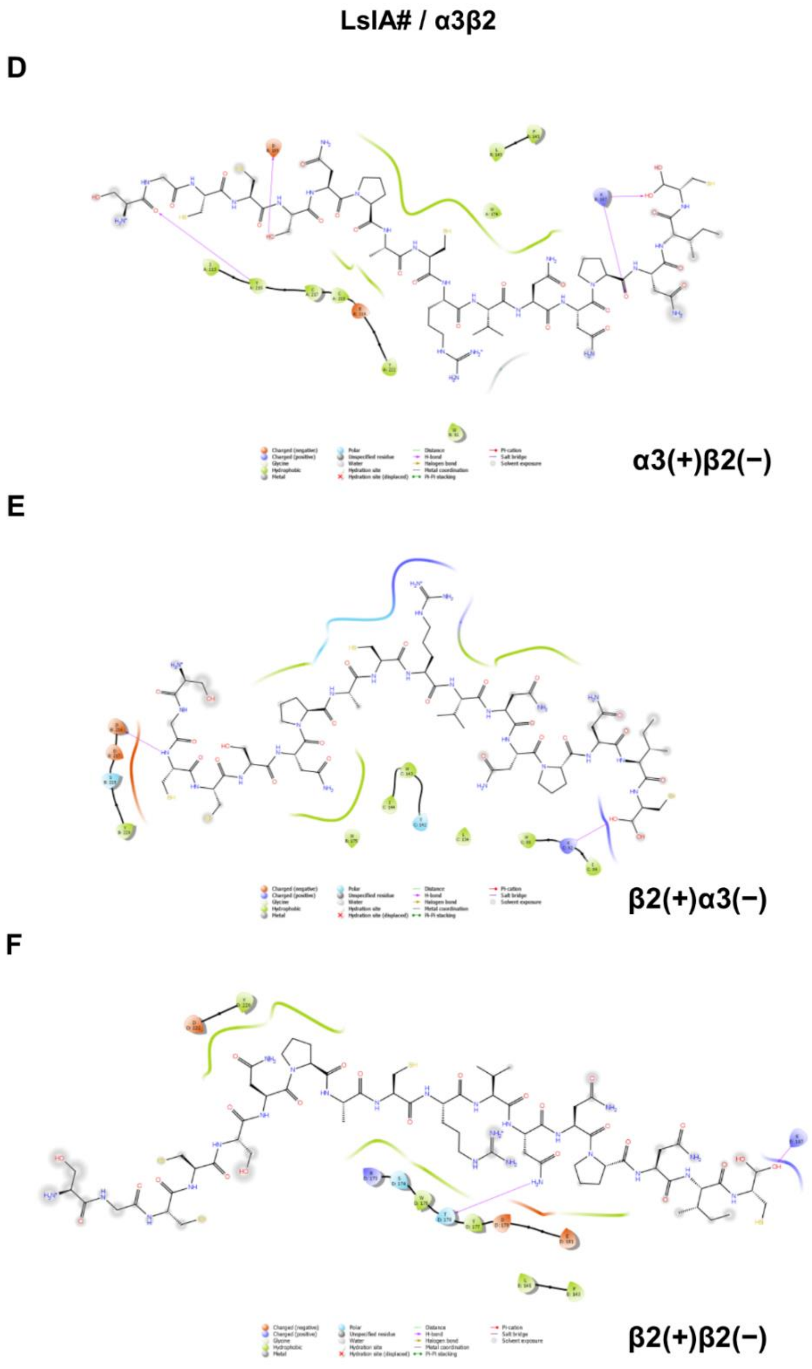

Figure S4. The α3β2 nAChR interface interactions with LsIA and LsIA#, respectively, according to the statistically significant variations of receptor contacts by C-terminal carboxylation. The top-ranking cluster (cluster #1) was chosen from the whole trajectory for each interface for ligand interaction analyses via Maestro (Schrödinger Release 2020-2) [1]. The structure of LsIA **(A, B** and **C)** /LsIA# **(D, E** and **F)** was selected based on cluster analysis in GROMACS packages. The observed hydrogen bond, hydrophobic and stacking interactions of LsIA/LsIA# with corresponding residues of α3β2 subtype are shown in the figure.

- LsIA and LsIA# intra-molecular interactions

To further explain the effects of the C-terminal amidation/carboxylation of LsIA on its interactions with α3β2 nAChR, we calculated the number of inter-atomic contacts and the atomic distances between the C-terminal of LsIA with other residues within the toxin, as well as between each residue of LsIA and the receptor at the α3(+)β2(–) and β2(+)α3(–) binding interfaces.

At α3(+)β2(–) interfaces, for LsIA#, the short distances (2.7 Å and 3.4Å) between carboxylated C-T (C17) and LsIA#-R10, allows an intramolecular salt bridge formed by C17 and R10 between them (Figure S5), whereas this kind of interaction is absent in LsIA. Figure S5A shows an illustrative graphic, whereas Figure S5B shows histograms of the C17-R10 distance, indicating the lower separation for LsIA#. These strong contacts also exist in LsIA# binding at β2(+)α3(–) interfaces, but are slightly reduced at β2(+)β2(–) interfaces (not shown), providing further support for the distinctiveness of the β2(+)β2(–) interface compared to the mixed-subunit interfaces, as noted in previous sections. In summary, this finding supports the distinct pattern at RMSF and "diff in RMSF" plots of chain I (β2(+)β2(–)), indicating higher rigidity of LsIA# versus LsIA may be partly due to enhanced internal salt bridges which restrict the toxin's mobility.

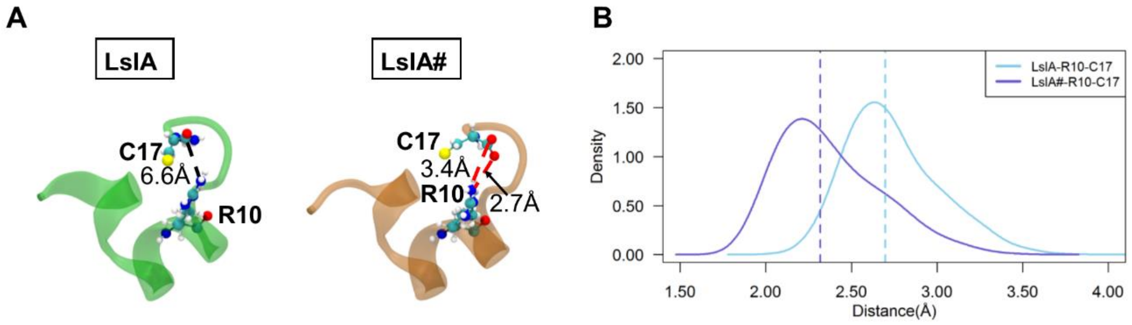

Figure S5. **Intra-molecular contacts between R10 and C17 of LsIA# versus LsIA binding at α3(+)β2(–) interfaces. (A)** Significant intra-molecular contacts formed within the LsIA# (transparent orange colour) versus the LsIA (transparent green colour), upon binding at α3(+)β2(–) interface. Important residues regarding intra-molecular interactions are shown in CPK form. The red dashed line represents the contacts that are much stronger in this form of LsIA/α3β2 complex. **(B)** The probability density function of distance (Å) between the coupled residues of LsIAs, and the coloured dash line demonstrates the median distance of the pairwise interactions.

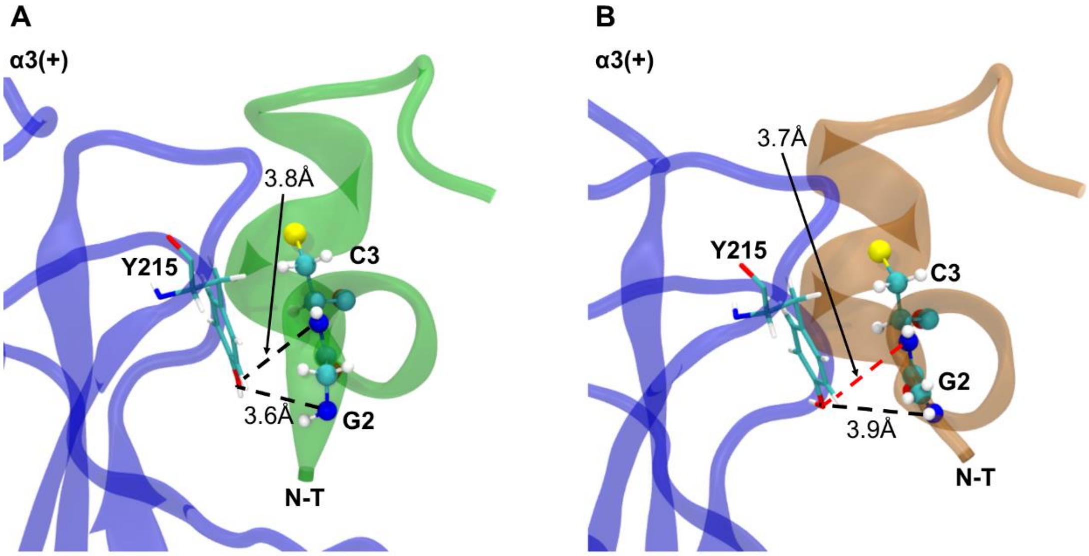

Figure S6. Interactions formed by LsIA-G2 and C3 with the receptor residues on the principal (+) face at α3(+)β2(–) interface of α3β2 nAChR. Binding conformation of LsIA **(A)** and LsIA# **(B)** at the (+) face with the pairwise interactions, which may substantially affect the binding affinity. The key residues involved in pairwise interactions on LsIAs are shown in CPK form, while the corresponding residues on the receptor are depicted with the Licorice form (a drawing format of graphic representation in VMD).

- Pairwise interactions weakly affect the binding affinity at α3(+)β2(–) interfaces

The pairwise interactions in both receptor-ligand complexes, namely S5-(+)D195, P7-(+)W174, and G2-(+)Y215, mainly contribute to stabilizing the binding of LsIA/LsIA# to the α3(+)β2(–) interfaces. These aromatic residues have also been suggested to play an important role in the binding of α-conotoxin ImI and ACh to human α7 and rat α3β2 nAChR, respectively, via forming cation-π interactions with ACh and ImI-R7 [2-4]. We observed persistent interactions between the aromatic residues on the α3(+) face and the conserved residues, C9 and P7, of LsIA in both LsIA and LsIA# anchored forms, such as P7-(+)W174 on the α3(+)β2(–) interfaces (Figures S4A, D and S7). The conserved proline exists in most α-conotoxins in loop 1 [5,6].

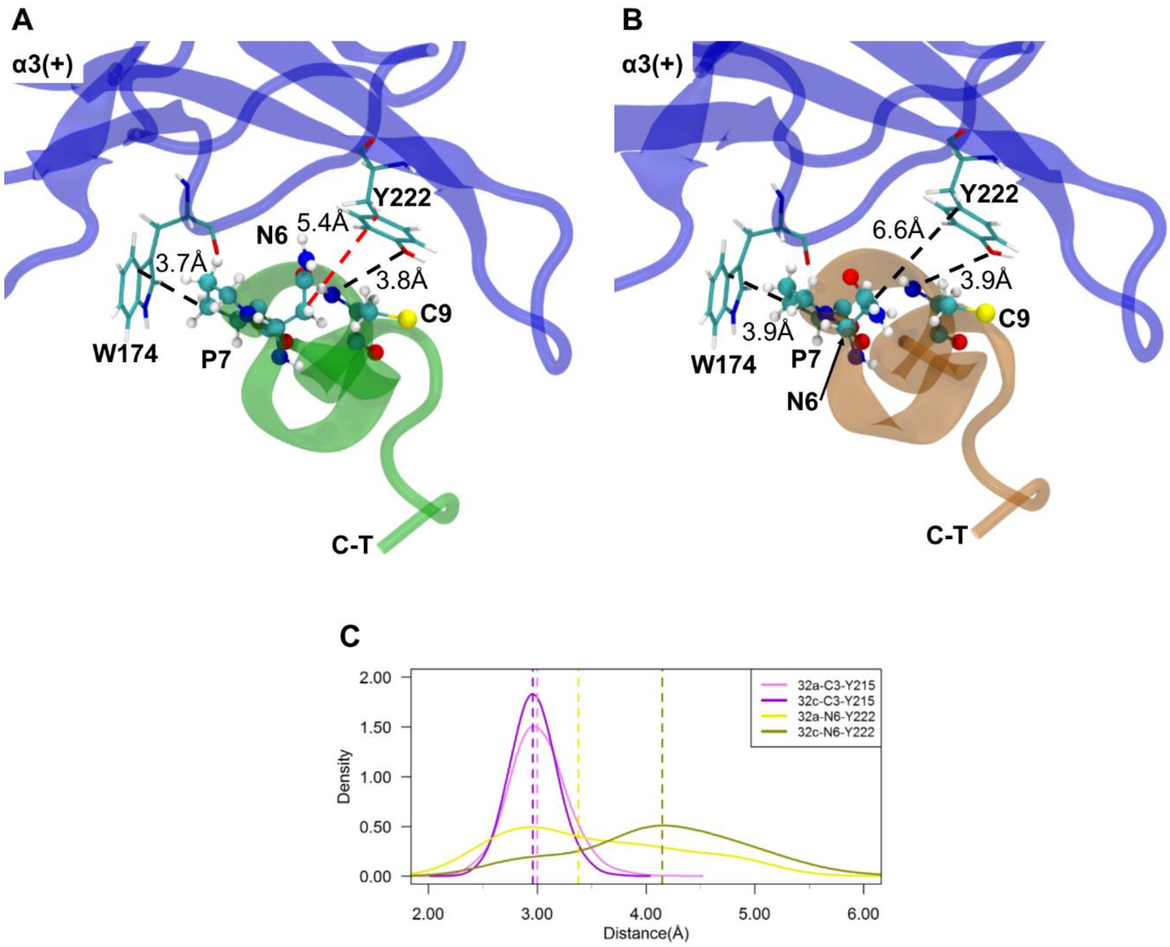

Figure S7. Interactions formed by LsIA-P7 and C9 with the receptor residues on the principal (+) face at *α*3(+)β2(–) interface. Binding mode of LsIA **(A)** and LsIA# **(B)** at the (+) face with the pairwise interactions which weakly affect the binding affinity. **(C)** The probability density function of distance (Å) between the coupled residues of bound forms. *32a* represents the LsIA bound type, whilst *32c* denotes the LsIA# bound complex.

- Pairwise interactions weakly affect the binding affinity at β2(+)*α*3(–) interfaces

At the principal β2(+) face, the conserved residues, P7 and C9 (not shown), and polar residue N6 of LsIA interact (Figure S8) in a similar manner to those at *α*3(+)β2(–) (Figure S7). Specifically, P7 exhibits higher contacts with aromatic residues, (+)W175 and (+)Y220 on β2(+) face for LsIA# compared to LsIA, whereas which establish relatively enhanced contacts with LsIA-A8. However, the differential β2 (+) contacts by N6, P7, A8 and C9 slightly affect the enhanced contacts by LsIA# at β2(+)*α*3(–) interfaces.

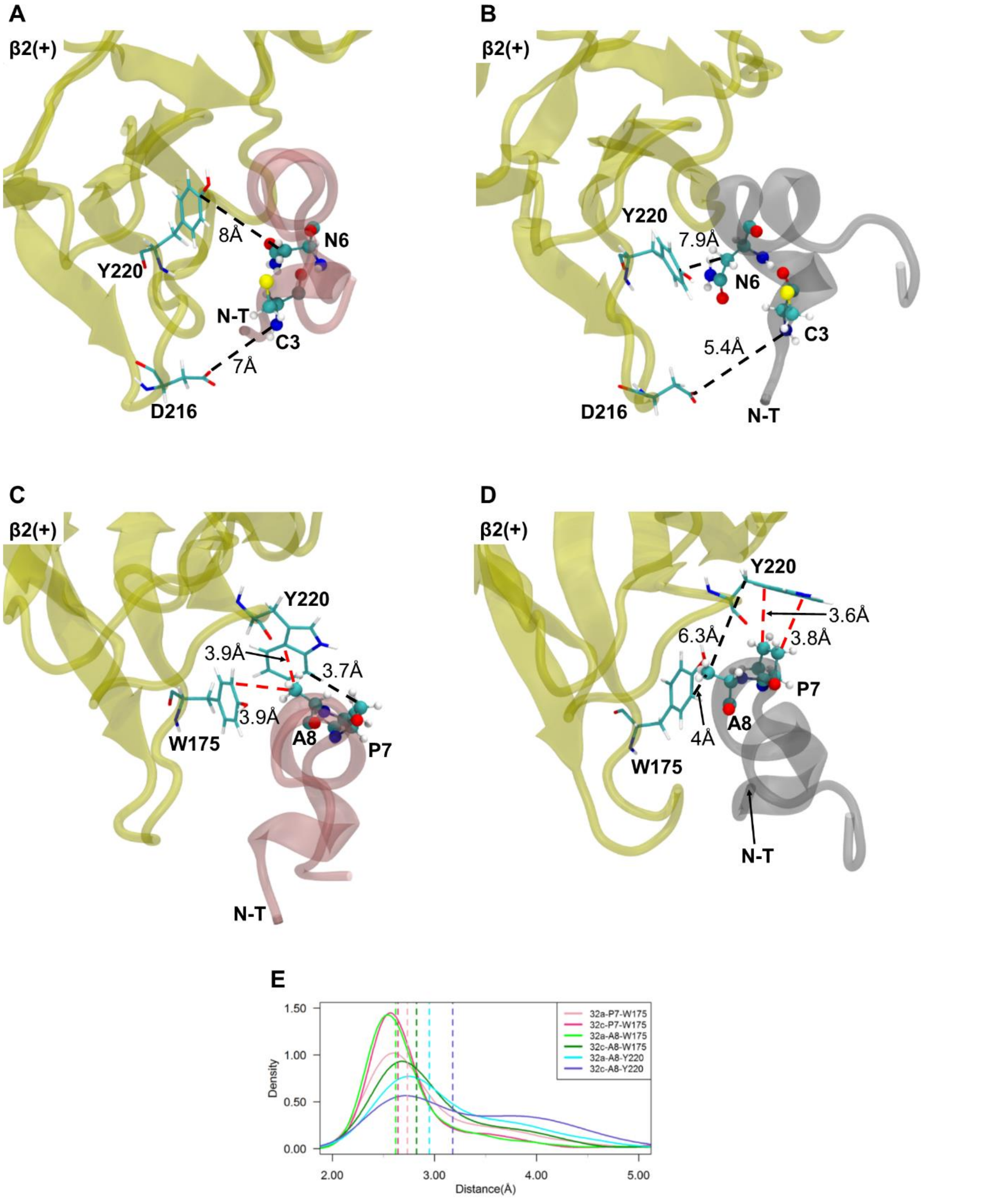

Figure S8. Interactions formed by LsIA-P7 and A8 with the receptor residues on the principal (+) face at β2(+)α3(–) interface of α3β2 nAChR. Binding conformation of LsIA **(A** and **C)** and LsIA# **(B** and **D)** at the (+) face with the pairwise interactions which weakly affect the binding affinity. The LsIA and LsIA# binding at the interfaces are shown in transparent pink and grey colours, respectively. **(E)** The probability density function of distance (Å) between the coupled residues of bound forms.

- Pairwise interactions at β2(+)β2(−) interface of α3β2 nAChR bound by LsIA

At the β2(+)β2(−) interface, the hydrogen bonds formed between β2(+)-Y177 and the non-conserved residue N12 of LsIA, reinforce the contacts with amidated LsIA bound complex relative to the LsIA# (Figure S3,S9). Apart from this, the contacts formed by β2(+)-Y220 on the β10 sheet of β2 subunit with LsIA#-C9, are also substantially reduced versus LsIA bound form in terms of van der Waals interactions. Notably, the homologous residues of α7 nAChR subunit corresponding to Y177 and Y220 of β2, have been suggested to form polar interactions with ImI-R7 via aromatic interactions [2,3] and related to the binding of α-conotoxin [7]. In addition, a hydrogen bonding contact formed by (+)D178 and N12 and the hydrophobic interactions between (+)W175 and P7, are slightly improved in the LsIA bound complex. Nevertheless, we observed few pairwise contacts with significant enhancement resulting from the C-terminal carboxylation of LsIA at this interface, possibly due to the loss of contacts with R10 and C17 of LsIA#. Interestingly, on the (−) face of the β2(+)β2(−) interface, the only apparent change in interactions occurs between P14 and (−)F143, compared with other pairwise interactions at α3(+)β2(−) and β2(+)α3(−) sites. The few variations between LsIA and LsIA# in contact with residues on (−) face at this site probably result from the absence of key distinguished receptor interactions by LsIA#-C17 and R10.

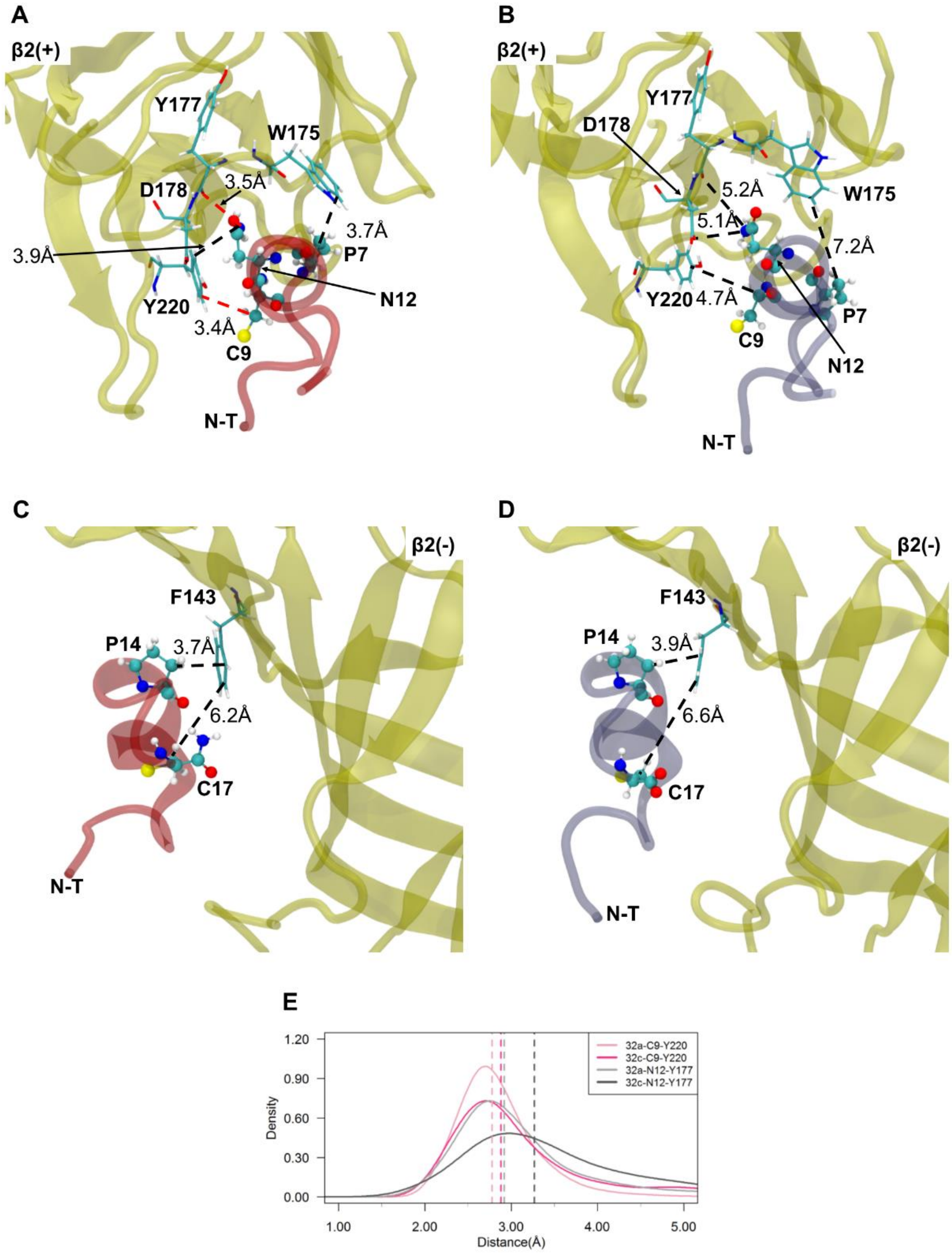

Figure S9. Important interactions formed by LsIA-C9 and N12 with the relative residues on β2(+)β2(–) interface of α3β2 nAChR. Binding conformation of LsIA **(A)** and LsIA# **(B)** at the (+) face with the pairwise interactions drastically affecting the binding affinity. Binding conformation of LsIA **(C)** and LsIA# **(D)** at the (–) face with the pairwise interactions. The LsIA and LsIA# binding at the interfaces are shown in transparent red and ice-blue colours, respectively. **(E)** The probability density function of distance (Å) between the important pairwise interactions of bound forms.

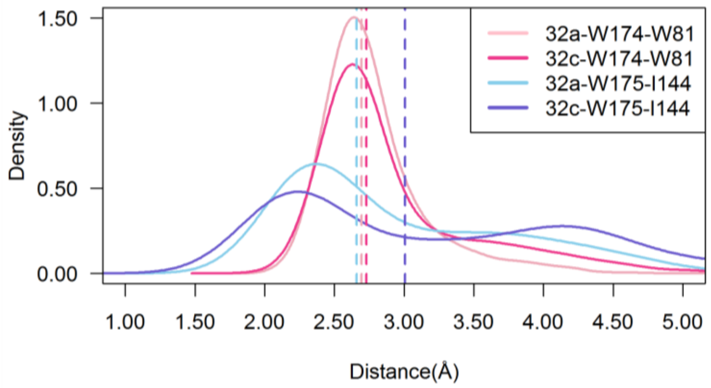

Figure S10. The distance of the inter-subunit interactions between the principal (+) and the accessory (–) face of α3β2 nAChR interfaces bound by LsIAs.

References

1. Schrödinger *Maestro (Version 2020-2)*, Schrodinger LLC: New York, NY, **2020**.
2. Dutertre, S.; Nicke, A.; Tyndall, J.D.A.; Lewis, R.J. Determination of α-conotoxin binding modes on neuronal nicotinic acetylcholine receptors. *J. Mol. Recognit.* **2004**, *17*, 339-347, doi:10.1002/jmr.683.
3. Ellison, M.; Gao, F.; Wang, H.-L.; Sine, S.M.; McIntosh, J.M.; Olivera, B.M. α-Conotoxins ImI and ImII target distinct regions of the human α7 nicotinic acetylcholine receptor and distinguish human nicotinic receptor subtypes. *Biochemistry* **2004**, *43*, 16019-16026, doi:10.1021/bi048918g.
4. Sambasivarao, S.V.; Roberts, J.; Bharadwaj, V.S.; Slingsby, J.G.; Rohleder, C.; Mallory, C.; Groome, J.R.; McDougal, O.M.; Maupin, C.M. Acetylcholine promotes binding of α-conotoxin MII at α3β2 nicotinic acetylcholine receptors. *ChemBioChem* **2014**, *15*, 413-424, doi:10.1002/cbic.201300577.
5. Armishaw, C.; Jensen, A.A.; Balle, T.; Clark, R.J.; Harpsøe, K.; Skonberg, C.; Liljefors, T.; Strømgaard, K. Rational design of α-conotoxin analogues targeting α7 nicotinic acetylcholine receptors: Improved antagonistic activity by incorporation of proline derivatives. *J. Biol. Chem.* **2009**, *284*, 9498-9512, doi:10.1074/jbc.M806136200.
6. Cartier, G.E.; Yoshikami, D.; Gray, W.R.; Luo, S.; Olivera, B.M.; McIntosh, J.M. A new α-conotoxin which targets α3β2 nicotinic acetylcholine receptors. *J. Biol. Chem.* **1996**, *271*, 7522-7528, doi:10.1074/jbc.271.13.7522.
7. Dutertre, S.; Lewis, R.J. Computational approaches to understand α-conotoxin interactions at neuronal nicotinic receptors. *Eur. J. Biochem.* **2004**, *271*, 2327-2334, doi:10.1111/j.1432-1033.2004.04147.x.
